# Comparative genomics of *Sympodiorosea* identifies genome evolution mediated through selective pressure on the metabolic gene repertoire

**DOI:** 10.1093/g3journal/jkag251

**Published:** 2026-09-21

**Authors:** Jacoby C Robinson, Aileen Berasategui, Quimi Vidaurre Montoya, Andre Rodrigues, Jeffrey Sosa-Calvo, Zoe Zimmerman, Yuliana Christoper, Hermógenes Fernández-Marín, Timothy D Read, Nicole M Gerardo

**Affiliations:** Department of Biology, Emory University, Atlanta, GA 30322, United States; Center for Biotechology, Genomics and Bionformatics, Virginia State University, Petersburg, VA 23806, United States; Amsterdam Institute for Life and Environment, Vrije Universiteit Amsterdam, Amsterdam 1081 HV, Noord-Holland, the Netherlands; Department of General and Applied Biology, São Paulo State University (UNESP), Institute of Biosciences, Rio Claro, São Paulo 13506-90, Brazil; Department of General and Applied Biology, São Paulo State University (UNESP), Institute of Biosciences, Rio Claro, São Paulo 13506-90, Brazil; Department of Biology, University of Central Florida, Orlando, FL 32816, United States; Department of Biology, Emory University, Atlanta, GA 30322, United States; División de Biodiversidad, Instituto de Investigaciones Científicas, Ciudad del Saber, Clayton, Panamá 0843-01103, Panamá; Facultad de Biociencias y Salud Pública, Universidad Especializada de las Américas (UDELAS), Extensión Veraguas 0901, República de Panamá; División de Biodiversidad, Instituto de Investigaciones Científicas, Ciudad del Saber, Clayton, Panamá 0843-01103, Panamá; Sistema Nacional de Investigación (SNI), SENACYT, Clayton, Panamá 0843-01103, Panamá; Division of Infectious Diseases, Department of Medicine, School of Medicine, Emory University, Atlanta, GA 30322, United States; Department of Biology, Emory University, Atlanta, GA 30322, United States

**Keywords:** *Sympodiorosea*, fungal comparative genomics, attine-ant symbiosis, *Hypocreales*

## Abstract

Biological interactions involving host-associated fungi are driven by chemistry shaped over evolutionary time. For antagonistic fungi, specialization often involves acquiring genes for novel secretions—including proteins and specialized metabolites—that mediate nutrient acquisition and host defense interactions. Here, we conducted a phylogenomic investigation of *Sympodiorosea*, ascomycetes commonly found in basal fungus-growing ant gardens. While *Sympodiorosea* and related genera (*Escovopsis*, *Luteomyces*, *Manidigitorum*, *Escovopsioides*) are canonically viewed as virulent mycoparasites, non-virulent species may also emerge within these attine-associated fungi. We explored genomic variation in *Sympodiorosea* to assess potential alternative lifestyles, focusing on the diversity and evolution of metabolic genes. Our study revealed a constrained selective landscape across the *Sympodiorosea* genome. However, outcomes of in vitro host interactions were diverse and predictable based on the antagonist's ant-species-of-origin, suggesting functional diversification. Phylogenomics indicated that gene gain/loss events in carbohydrate-active enzymes and specialized metabolism drive this diversification. Although selection acts intensely on metabolism-related genes overall, specific metabolic genes experience diversifying selection, indicating their critical role in host associations. Furthermore, *Sympodiorosea* exhibits protease gene expansions and contractions discordant with a strictly mycoparasitic lifestyle, suggesting either an alternative lifestyle within ant gardens or recent evolution from other niches. These results provide novel insights into fungal genome evolution and the chemistry of the fungus-growing ant symbiosis.

## Introduction

Fungal parasites and antagonists vary tremendously in terms of their host range and the modes used to identify, contact, and infect hosts. Much of their evolutionary success is reliant on their capacity to synthesize and secrete a diverse array of chemical compounds that mediate interactions with their hosts and with competitors (de Bekker et al. 2017; Keller 2019; Case et al. 2022). While the ecological, economic, and epidemiological importance of some fungal metabolites has been elucidated, less is known about how metabolic diversity across fungi evolves. Our gap in understanding is magnified by the difficulty of culturing many fungal species (Meyer et al. 2016) and the polynucleated genomic structure of some fungi, which makes them less amenable to genomic and computational approaches than other microbes (Kooij et al. 2015; Meyer et al. 2016). Furthermore, a multipronged approach that combines experimental and comparative approaches is needed to generate a mechanistic model of genome evolution through the lens of the metabolic gene repertoire an organism contains. Here, to gain insight into metabolic evolution of antagonistic fungi, we used a natural system with an ancient history of association that is well-suited for investigation of how metabolic genes evolve (de Man et al. 2016; Gotting et al. 2022; Berasategui et al. 2024). Ascomycete fungi in the genus *Sympodiorosea* (*Hypocreales*: *Hypocreaceae*) have only ever been found in association with gardens of fungus-growing attine ants belonging to the “lower attines” and are considered parasites to the ants’ basidiomycete cultivars (i.e. the fungi that the ants cultivate for food) (Currie et al. 1999a; Bot et al. 2001; Currie and Stuart 2001; Gerardo et al. 2004; Augustin et al. 2017). The cultivars and ants have an array of defenses that can reduce the impacts of fungal antagonists (Currie et al. 1999b, 2006; Heine et al. 2018; Kyle et al. 2023), raising the question of how *Sympodiorosea* can persist in seemingly healthy gardens in nature. These defenses, as well as interactions with microbial competitors within the gardens, likely have impacted *Sympodiorosea* evolution. With the identification of bioactive metabolites in closely related fungi (Heine et al. 2018) and recent broad-scale genomic investigations of metabolic potential of *Sympodiorosea* and closely related fungi associated with fungus-growing ants (Gotting et al. 2022; Berasategui et al. 2024), our primary goals were to assess how metabolic gene diversity within the *Sympodiorosea* genus varies across strains and how selection shapes this diversity within the context of the antagonistic interaction between *Sympodiorosea*, the ants’ cultivars, and, as a consequence, the ants themselves.

The fungus-growing ant system is well-suited to investigate the impact of complex symbiotic interactions on fungal evolution (Mueller et al. 1998; Currie et al. 2003; Gerardo et al. 2004). To date, there are 5 described genera in the family *Hypocreaceae* of putatively parasitic microfungi associated with fungus-growing ants (Osti and Rodrigues 2018; Montoya et al. 2021; da Cruz et al. 2026). These include the well-described genus *Escovopsis*, *Escovopsiodes*, and the newly described genera *Sympodiorosea*, *Luteomyces*, and *Manidigitorum* (Montoya et al. 2021, 2023; da Cruz et al. 2026), which were split from *Escovopsis*. For these ascomycetes, interactions with the ants’ gardens’ microbial communities are likely mediated by chemistry and secretions produced by the cultivated fungi (Gerardo et al. 2006; de Oliveira et al. 2023; Goes et al. 2024), as well as those produced by other bacteria and fungi commonly found in gardens (Currie et al. 1999b, 2006). Recent genomic analyses described functional variation in regions related to metabolism across these genera (Gotting et al. 2022; Berasategui et al. 2024), revealing that secondary metabolism broadly differs across these taxa. This suggests that their chemistry is evolving according to genus-specific selective pressures. For a symbiotic fungus, genes encoding metabolic secretions are likely to experience significant selection due to the necessity to produce compounds critical for colonization, signaling, defense, and nutrient acquisition (Girard et al. 2013; Barrett et al. 2020; Wang et al. 2022; Jia et al. 2023; Saciloto-de-Oliveira et al. 2023) and due to the energetic costs associated with producing these secretions. This suggests that distributions of secondary metabolites may reveal the history of adaptation to specific niches or hosts, and that signatures of selection on the genes encoding for critical metabolites may be different than those of other genes not evolving in response to dynamic species interactions.

For *Sympodiorosea* spp., evolution may be shaped by interactions with the ants, the fungi cultivated by the ants, and by other microbes in the ants’ gardens. *Sympodiorosea* is commonly isolated from the gardens of a subset of the attine ants, including ants in the genera *Cyphomyrmex*, *Myrmicocrypta*, and *Mycetophylax* (Custodio and Rodrigues 2019; Montoya et al. 2021). *Cyphomyrmex* obligately cultivate basidiomycete fungi in both filamentous and yeast forms and are geographically distributed across central America, reaching from southern parts of the United States to the southern tip of Argentina (Mueller et al. 1998; Currie et al. 2003; Schultz and Brady 2008). Previous work within the *Cyphomyrmex* association has demonstrated considerable diversity in interaction outcomes between *Sympodiorosea* and cultivar strains (Birnbaum and Gerardo 2016). Acting as an antagonist to the cultivars, *Sympodiorosea* can recognize and overgrow cultivars grown by the native ant species from whose gardens they were isolated; less often, they can overcome defenses of non-native cultivars grown by other ant species (Gerardo et al. 2004; Birnbaum and Gerardo 2016). These results indicate genus- and species-level mechanisms of specialization for the antagonistic fungi. Consistent with these findings, previous work using marker genes revealed that more closely related *Sympodiorosea* are found in *Cyphomyrmex* colonies with more closely related cultivars (Gerardo et al. 2004; Birnbaum and Gerardo 2016). Specifically, while *Cyphomyrmex longiscapus* is closely related and ecologically similar to *muelleri muelleri*, the 2 ant species cultivate fungi from separate clades (“*Leucocoprinus* clade 1” and “*Leucocoprinus* clade 2”), whereas *Cyphomyrmex costatus* are not found in the same habitats and are more distantly related yet cultivate fungi genetically similar to the cultivars of *C. muelleri* from “clade 2” (Green et al. 2002; Mehdiabadi et al. 2012; Schultz et al. 2024). Diversification of *Sympodiorosea*, based on marker genes, in relation to these ants and their cultivars suggests that the *Sympodiorosea* in *C. muelleri* and *C. costatus* colonies are, in general, more similar to each other than they are to *Sympodiorosea* from *C. longiscapus* colonies, though switches do occur (Gerardo et al. 2004; Birnbaum and Gerardo 2016). This has never been studied in relation to genes that may be functionally important for interactions within the context of association with particular cultivars and their ant farmers.

Here, we use comparative genomics to describe the phylogenomic landscape of *Sympodiorosea*. After establishing a whole genome-based phylogeny, we assess diversification across the *Sympodiorosea* genus, specifically focusing on genomic regions related to primary and secondary metabolism. Our results indicate that variation in metabolic genes is constrained by purifying selection and demonstrate that, at the genomic scale, *Sympodiorosea* likely are evolving in response to interactions with both the cultivars and the ants. These results indicate a further need to explore the chemical mechanisms that underpin interactions between associated fungi.

## Materials and methods

### Assessing morphology and in vitro host overgrowth

Twenty-two isolates of *Sympodiorosea* and 9 cultivars (i.e. the fungi cultivated by ants for food) were obtained from a culture collection maintained at Emory University (Supplementary Tables 1 and 2). The 22 *Sympodiorosea* comprise 11 strains isolated from *C. longiscapus* colonies, 3 strains isolated from *C. costatus* colonies, 3 strains isolated from *C. muelleri* colonies, 1 strain isolated from novel species of *Cyphomyrmex* attines (*C*. sp. nov.), 2 strains isolated from a colony of an unknown species of *Cyphomyrmex* (*C.* sp.), 1 strain isolated from a *Myrmicocrypta ednaella* colony, and 1 strain isolated from an unknown attine species (Supplementary Table 1).

Samples were revived from 10% glycerol stocks and stored at room temperature. All samples were maintained on potato dextrose agar (PDA) and transferred once Petri dishes were overgrown or every 14 d, depending on growth rate. To assess gross in vitro morphology, *Sympodiorosea* were grown in monoculture. The cultures were incubated at 25 °C and photographed every 3 d up to the 28-d mark.

To assess in vitro antagonism directed at cultivar hosts, we set up pairwise bioassays of the 22 *Sympodiorosea* strains with the 9 cultivar strains (Supplementary Table 2). The cultivars included 6 isolated from colonies of ants in the genus *Cyphomyrmex*. These cultivars are all in the family *Agaricaceae* (order Agaricales) and represent 2 main clades of *Leucocoprinus* cultivars grown by lower attines (Mueller et al. 1998; Schultz et al. 2024). Specifically, the 3 strains isolated from *C. longiscapus* colonies are from “Leucocoprinus clade 1,” and the 3 strains isolated from *C. muelleri* and *C. costatus* colonies are from “*Leucocoprinus* clade 2” (Schultz et al. 2024). The remaining 3 cultivar strains were isolated from *Apterostigma* spp. colonies. These, *Mymrmecopterula* sp., “coral fungus cultivars,” in the family *Pterulaceae* (order Agaricales), are distantly related to the lower fungal cultivars (Schultz et al. 2024). For each combination of cultivars and *Sympodiorosea*, cultivars were plated in triplicate, ∼5 mm from the edge of a 60 mm Petri dish containing PDA, by transferring a 5 mm plug of fresh mycelium on PDA from a Petri dish. They were allowed to grow for 7 d at 25 °C. At day 7, *Sympodiorosea* were plated centrally in the same manner. Experimental plates were incubated at 25 °C and photographed every 3 d up to the 28-d mark. Images of *Sympodiorosea*-cultivar interactions were analyzed in ImageJ v1.53 (Schneider et al. 2012). Interaction fidelity was measured through a binary, where “1” indicates that *Sympodiorosea* overgrew the cultivar and “0” indicates that *Sympodiorosea* was unsuccessful in overgrowing the cultivar because: (i) the cultivar inhibited *Sympodiorosea* growth at a distance; (ii) *Sympodiorosea* was inhibited at the point of contact; or (iii) *Sympodiorosea* failed to invade the cultivar hyphae in the event contact occurred (Christopher et al. 2021).

### Whole-genome sequencing of *Sympodiorosea*

Genomic DNA extractions were performed for each of the *Sympodiorosea* samples used in bioassay experiments. Strains were first grown on PDA for 7 d, at which point a small plug of each strain was transferred to a new PDA plate to ensure monoclonal conditions. After an additional 4 d, fresh mycelia from each of the strains were gently scraped off the agar using sterilized spatulas. For each strain, 500 µL of fresh mycelia was collected in 1.5 mL Eppendorf tubes containing 500 µL ddH_2_O and centrifuged at 13,000 RPM for 5 min. Care was taken to ensure PDA did not dislodge into the mycelial suspension. The supernatant was removed, and 200 µL lysis buffer (recipe in Supplementary Methods and Figures) and 100 µL of microbeads were added. Two hundred microliters of phenol:chloroform:isoamyl alcohol (25:24:1) were added to the suspension before vortexing for 30 min. Two hundred microliters of TE buffer were added to each tube before centrifugation at 13,000 RPM for 5 min. The aqueous layer was removed, and each sample underwent a second extraction with 400 µL phenol:chloroform:isoamyl alcohol. DNA was precipitated overnight in 100% methanol at 4C, dried, and resuspended in 50 µL ddH_2_O. All samples were sequenced through OmegaBioservices in 2021 using the Illumina NovaSeq 6000 platform. Manufacturer protocols were used to prepare samples for PE 2 × 150 short-read sequencing using the NovaSeq 6000 SP Reagent Kit (Modi et al. 2021).

### Generation of ultra-conserved element sequences

The methods employed to generate the ultra-conserved element (UCE) fungal phylogenomic data follow the methods in Schultz et al (2024). Laboratory work (DNA extraction, library preparation, and targeted enrichment) was carried out in the Laboratories of Analytical Biology (LAB), National Museum of Natural History (NMNH), Smithsonian Institution.

#### DNA extraction

DNA was extracted from fungal tissues cultured in the lab. A small fragment of fungal tissue was placed into a 2 mL tube with stainless-steel beads for tissue disruption at 25 Hz for 30 s employing a TissueLyser II (Qiagen). DNA of fungal samples was extracted using the Qiagen DNeasy Plant Mini Kit (Qiagen, Valencia, CA, United States), following the recommendations of the manufacturer, except for conducting lysis overnight in a dry bath shaker set at 65 °C and 500 RPM, and eluting the extracted DNA from the spin-collection columns in 2 steps, each using 50 μL of ddH_2_O.

#### Shearing and library preparation

DNA extractions were quantified with a Qubit 3.0 Fluorometer employing the High Sensitivity Kit (Thermo Fisher Scientific, Inc.) using 1 μL of DNA template. Prior to library preparation, 50 ng of DNA template was sheared to an average fragment length of 300 to 600 bp using a Qsonica Q800R3 Sonicator (Qsonica LLC, Newton, CT, United States) for 60 s, 25% amplitude, and shearing cycles of 10 s.

Library preparation was carried out using the Kapa Hyper Prep Library Kit (Kapa Biosystems, Inc., Wilmington, MA, United States) as described in Faircloth et al. (2015) with the iTru Adapters protocol. We implemented all magnetic bead clean-up steps (Fisher et al. 2011) using a SPRI substitute (Rohland and Reich 2012) as described in Faircloth et al. (2015). Dual-indexing TruSeq adapters (Faircloth and Glenn 2012; Glenn et al. 2019) were used for ligation and followed by PCR amplification of 15 μL of the library using the KAPA HiFi Ready Mix (Kapa Biosystems, Inc., Wilmington, MA, United States) as described in Schultz et al (2024). The resulting DNA product was purified using 1.2× Kapa Pure Beads and rehydrated in 23 μL of elution buffer (EB, pH = 8). Individual libraries were then quantified using 2 μL of library product in a Qubit 3.0 Fluorometer using the Broad Range Kit.

#### Sample pooling and target enrichment of libraries

Post-PCR libraries were pooled together (10 libraries) at equimolar concentrations into a single pool. Pool concentration was adjusted to 72 ng/μL by drying samples (until all liquid evaporated) in a vacuum centrifuge at 60 °C. Dried pools were resuspended in nuclease-free water at a previously estimated volume for the pool. The pool concentration was calculated using a Qubit 3.0 Fluorometer with the Broad Range Kit. The final concentration of the pre-enrichment pool was 58.1 ng/μL.

The resulting fungal pool was enriched for 24 h employing a custom UCE probe set (7,736 baits targeting 2,121 conserved loci) for Agaricales, at an incubation temperature of 65 °C. Enrichment, bead-cleaning, and PCR reaction procedures followed (Borowiec 2019; Schultz et al. 2024). The enriched pool was purified using 1.0× Kapa Pure Beads and rehydrated in 22 μL EB. The final cleaned enriched pool was quantified using 2 μL with the Qubit 3.0 Fluorometer and the Broad Range Kit.

The final enriched pool was sent to the Admera Health Biopharma Services (NJ, United States) for quality control and sequencing on an Illumina NovaSeq X Plus 10B (125 cycle paired-end sequencing) run. Newly generated fungal sequence data for this study are deposited in the NCBI Sequence Read Archive (SRA) under BioProject ID PRJNA1502419, SRA numbers SAMN61974365–SAMN61974374. A list of newly generated samples and samples from previous studies are detailed in Supplementary Table 4.

#### Processing of UCE sequence data

Demultiplexed fungal raw sequences were trimmed for adapter contamination and low-quality bases using Illumiprocessor v.2.0.6 (Del Fabbro et al. 2012; Lohse et al. 2012; Faircloth 2013) modified by R. Dikow and P. Frandsen to use trim_galore (v0.4.1 (Krueger et al. 2021)), with the --retain_unpaired option enabled. The program SPAdes v.3.14 (Bankevich et al. 2012; Nurk et al. 2013) was employed to assemble the clean reads into contigs. Newly generated UCE sequences were identified using the Phyluce script phyluce_assembly_match_contigs_to_probes (Phyluce v1.6.7, Faircloth 2016) with the options --min-coverage and --min-identity set at 50 and 80, respectively.

Newly generated UCE fungal sequences were combined with 99 fungal sequences (both free-living and ant-associated fungal cultivars) from Schultz et al. (2024) downloaded from NCBI SRA and were further processed using a series of scripts available in the Phyluce package v1.6.7 (Faircloth 2016) and following the methods described in Branstetter et al. (2017a, b); Li et al. (2018), and Hanisch et al. (2022). Each UCE locus was aligned using MAFFT v7.407 (Katoh and Standley 2013) employing the L-INS-I option, which is known to produce highly accurate alignments (Katoh et al. 2005; Katoh and Standley 2013). The program GBLOCKS v.091b (Talavera and Castresana 2007) was used with relaxed settings (-b1 = 0.5 -b2 = 0.5 -b3 = 12 -b4 = 7) to remove poorly aligned regions in each UCE locus. We selected those UCE loci with at least 70% of the taxa represented (i.e. 76 out of 109 taxa) using the Phyluce script “phyluce_align_get_only_loci_with_min_taxa.” Locus names were removed from the taxon names using the Phyluce script “phyluce_align_remove_locus_name_from_nexus_lines,” and the resulting UCE loci were concatenated into a data matrix using the Phyluce script “phyluce_align_format_next_files_for_raxml” for downstream analysis. The resulting fungal data matrix consisted of 109 taxa (including 102 ant-cultivated fungi) and 190,741 nucleotides representing 252 UCE loci.

#### Analyses of UCE data

The Python program SPRUCEUP (Borowiec 2019) was used to remove poorly sequenced and/or poorly aligned sequence fragments from individual taxa for each locus. For the fungus alignment, we set the window size to 25 sites and the number of overlapping sites between sliding windows to 15, and we used a global cutoff of 0.99 for distance outliers. We conducted additional manual cutoffs: FUN1629_NGL220_AGH020522_03,0.35; FUN1630_NGL239_AS020605_01,0.65; FUN1631_NGL244_CC020302_04,0.60; FUN1632_NGL255_JS020524_12,0.75; FUN1633_NGL263_JS100417_09,0.65; FUN1635_NGL292_NMG030627_02,0.58; FUN1638_NGL347_UGM020531_01,0.75.

#### UCE data partitioning

Analyses were performed using the SPRUCEUP-trimmed alignment under the Sliding Window Site Characteristic based on Entropy method (SWSC-EN) (Tagliacollo and Lanfear 2018) partition scheme, which addresses UCE properties to identify, for each locus, the highly conserved core region and its 2 variable flanking regions (Faircloth et al. 2012) in order to create partitions. The SWSCEN algorithm identified 753 UCE subsets. Following this step, the best partitioning scheme and the best-fitting model of evolution was identified by merging the resulting subsets using ModelFinder (Kalyaanamoorthy et al. 2017) as implemented in IQ-TREE v.2.1.2 (Minh et al. 2020) with the option -m MF+MERGE, the fast relaxed -rclusterf algorithm (Lanfear et al. 2017) set to 10, which compares the top 10% of the resulting partitioning schemes with the corrected Akaike information criterion. The resulting best partitioning scheme consisted of 426 partitions.

#### UCE maximum-likelihood analyses

Maximum-likelihood (ML) analyses of the SPRUCEUP-trimmed fungal alignment were conducted in IQ-TREE v.2.1.2 (Chernomor et al. 2016; Minh et al. 2020) and used the partition scheme described above. Branch support was estimated using the ultrafast bootstrap (Hoang et al. 2018) and the SH-like approximation likelihood ratio test (Guindon et al. 2010), both set at 1,000 replicates, and other options set at the default values.

Analyses were conducted on the Smithsonian High Performance Cluster (SI/HPC), Smithsonian Institution (https://doi.org/10.25572/SIHPC).

### Genome assembly

We used Trimmomatic (v.0.39) to both trim adapter content and filter all reads for quality using “MINLEN”=36. High-quality reads were assembled de novo using SPAdes v.3.15.5 (Bankevich et al. 2012), with the “--fungus” and “--careful” flags. BUSCO (Benchmarking Universal Single-Copy Orthologs) (Simão et al. 2015) was used to assess assembly quality by searching against the “Ascomycota_odb10” universal single-copy ortholog database. All samples analyzed showed a “completeness” score >95%, indicating high quality assemblies with low levels of duplication or fragmentation (Supplementary Table 1).

### Functional annotation

Gene prediction and annotation were performed using the Funannotate (v.1.8.13) pipeline. Assembled contigs were prepared for gene prediction using the Funannotate “clean” command under default settings, whereby contigs shorter than 500 bp were removed. Repeats were identified and soft-masked with tantan (Frith 2011). Soft-masked genomes underwent gene prediction by first aligning proteomes from *Trichoderma* sp., *Cladobotryum* sp., *Hypomyces rosellus*, and *H. perniciosus* using DIAMOND/Exonerate. PASA (Do et al. 2024) gene models were used to train/run 3 gene prediction tools: Augustus (Stanke and Morgenstern 2005), SNAP (Bromberg and Rost 2007), and GlimmerHMM (Delcher et al. 1999). The joint predictions were then passed through EvidenceModeler to provide a consensus prediction (Haas et al. 2008).

We used BlastP searches against the Uniprot/SwissProt and MEROPS protein databases to generate functional annotations and identify protease and protease inhibitors, respectively (Altschul et al. 1990). Functional annotations fed through InterProScan5 (Jones et al. 2014) produced putative protein domains and sites as well as Gene Ontology (GO) terms with subsequent alignments inferred by searching against the Eggnog orthology database using emapper v3 (Cantalapiedra et al. 2021). Biosynthetic gene cluster (BGC) predictions were assigned using antiSMASH fungal v6 (Blin et al. 2021) with all predictive tools under relaxed parameters. Finally, carbohydrate-active enzymes (CAZymes) and their putative substrates were annotated using the dbCAN3 server (Zheng et al. 2023) with HMMER v3.3 (Finn et al. 2011).

### Phylogenetic reconstruction and genetic distance

The phylogenetic relationship of *Sympodiorosea* and their close relatives was reconstructed using the BUSCO phylogenomics pipeline (McGowan and Fitzpatrick 2020). The analysis included 46 publicly available proteomes (6 additional *Sympodiorosea* spp., 12 *Escovopsis* spp., 5 *Luteomyces* spp., 11 *Trichoderma* spp., 5 *Fusarium* spp., 2 *Hypomyces* spp., 1 *Cordyceps militaris*, and 1 *Cladobotryum* sp.) (Supplementary Table 3). The busco_phylogenomics pipeline first identified shared, single-copy orthologs using the BUSCO v5 “Hypoceales_odb10” lineage database. Protein sequences were aligned using MUSCLE (Edgar 2004), and alignments were trimmed with TrimAl (Capella-Gutiérrez et al. 2009). Trimmed alignments were concatenated into a supermatrix, and the alignment was partitioned by ModelFinder, which was allowed to infer the best model of evolution (Kalyaanamoorthy et al. 2017). The partitioned alignment was used as input for IQTree (Nguyen et al. 2015) to generate the phylogeny using the fast bootstrap flag “-B 1000” with *Cladobrotryum* spp., *Hypomyces rosellum*, and *H. perniciousus* as outgroups.

To place our sequences within a larger context of antagonists associated with fungus-growing ants, we generated a maximum-likelihood phylogeny using elongation factor 1-alpha (tef1-alpha) gene sequences via the NGphylogeny platform (Lemoine et al. 2019). We included 157 total samples spanning *Escovopsis*, *Luteomyces*, *Escovopsioides*, and *Sympodiorosea* (Supplementary Tables 1, 3 and 4). Sequences were aligned using MAFFT (Katoh and Standley 2013) with default settings, and phylogenetic informative sites were selected via BMGE (Criscuolo and Gribaldo 2010). The phylogeny was estimated with PhyML with Smart Model Selection (Guindon et al. 2010) using default settings, and branch support was based on SH-like aLRT branch support. Phylogenetic and phylogenomic trees were visualized using the Interactive Tree Of Life (iTOL v5) (Letunic and Bork 2021).

Additionally, we tested for phylogenetic signal of the host cultivar and ant associate using the PhyloSignal package in R. We tested whether ant species of origin correlates with *Sympodiorosea* relatedness and, similarly, whether cultivar host correlates with relatedness between *Sympodiorosea*. A consensus tree was combined with tip data related to the native ant species cultivating the gardens from which our 22 genome-sequenced strains and 4 publicly available *Sympodiorosea* genomes were isolated.

For the 22 *Sympodiorosea* strains sequenced as part of this work, average nucleotide identity values were calculated using PyANI (v0.2.10) under default settings. We used MUMmer (NUCmer) to align our 22 sequenced genomes. Pairwise sequence alignment was used to calculate the percentage identity between 2 given samples.

### Comparisons of metabolic gene content

Given that these strains vary in terms of their ability to contact and overgrow cultivar hosts in vitro, we investigated differences in the genes underlying substrate use, both of which could influence host use. Consensus gene predictions of CAZymes and proteases were extracted based upon transcriptome evidence from *Escovopsis weberi* as well as Diamond/BlastP annotations. The presence/absence data were used as input for principal component analysis and heatmap visualization. Principal component analyses were carried out with the function “prcomp” available in the base R stats package and visualized with ggplot2 (Wickham 2016). All heatmaps in our analyses were generated using the R package pheatmap (Kolde 2010).

In addition, putative functional annotations of CAZymes and proteases were converted to counts, and, subsequently, we determined a ratio of these for each genome (CAZyme:protease). These ratios were placed in the larger context of alternative fungal lifestyles by scraping data from Ahrendt et al. (2018) using WebPlotDigitizer v.4.6 (Ahrendt et al. 2018).

For BGCs, we used BiG-SCAPE to generate sequence similarity matrices by grouping gene cluster families (GCFs) based on sequence similarity and homology, with a cutoff score of 0.5. In the matrix, GCF presence is indicated by “1” and absence by “0.” GCF presence/absence was visualized with a heatmap using R. Exemplar BGCs that putatively encode known metabolites from the closely related species *E. weberi* were selected for synteny analysis using Clinker (Gilchrist and Chooi 2021) to understand how metabolic potential may be shaped at the locus level. From the putative predictions, we sought to identify known clusters by searching against the MIBIG database v3.1, which is composed of 2,502 secondary metabolite clusters, by using the flag “mibig” in the bigscape.py command.

### Linking in vitro overgrowth to genomic variation

To test for association between variation in overgrowth, metabolic gene content, and phylogenetic relatedness, we used the command “vegdist” from the R package “Vegan” (Oksanen et al. 2026) to generate dissimilarity matrices of Euclidean distance based on host-use and *Sympodiorosea* ANI, as well as protease, CAZyme, and BGC presence/absence. We used Spearman's correlation coefficients when using the ANI dissimilarity matrix in Mantel tests of association. Otherwise, tests were conducted using Kendall's correlation coefficient due to the nature of presence/absence data.

### Population genomics and selection

To understand the role of selection in diversification of *Sympodiorosea*, we calculated the ratio of nonsynonymous mutations (dN) to synonymous mutations (dS) for each ortholog that was previously assigned a GO term. GO enrichment analyses were executed for our 22 sequenced strains using GenBank-formatted annotations supplied to “Funannotate compare” with the flag “--run_dN/dS estimate” using the “M0” null model from codeml (Yang 2007). The M0 model effectively normalizes the dN/dS across all sites and branches for a particular gene, resulting in values ranging between 0 and 1. The resulting values are both relative and conservative estimates of selection, because, to detect diversifying selection, it would need to act intensely within a few samples or moderately across all samples. Our data composed of highly similar genomes further reduces the likelihood that diversifying selection can be detected, as sufficient time must pass for orthologous genes to acquire identifiable differences in their coding sequences. Ultimately, we seek to identify genes that are under diversifying selection and that may contribute to the coevolutionary relationship between *Sympodiorosea* and the other organisms within the fungus-growing ant symbiosis.

To evaluate signatures of selection across sequenced genomes, we implemented the free-branch model as a conservative estimate of selection, where selection is assumed to be consistent across all sites along all branches (0 < *θ* < 1). The resulting values are relative in that they are constrained between 0 and 1. Each ortholog in each genome was then assigned a value corresponding to the calculated ratio of dN to dS (hereafter referred to as dN/dS). We then randomly sampled 5,000 of the 6,027 orthologous genes to understand the distribution of dN/dS values. Randomly sampled values were subjected to the Shapiro–Wilk test of normality. Visual inspection as well as statistical evidence indicated our data were Poisson distributed. We calculated the mean, variance, and standard deviation (SD) of dN/dS values. We set a threshold of 3 SD (*λ* = 0.42) from the mean as the threshold to identify genes that differentially accumulate variation relative to all orthologous genes. This cutoff is in line with other studies of fungal pathogens/parasites that reported dN/dS ratios ranging between 0.1 and 0.4 for core and accessory genes (Cheng et al. 2015; Olarte et al. 2019; Petersen et al. 2023).

We then selected orthologous protein-encoding genes identified as the core proteome in our strains with OrthoFinder and Cluster of Orthologous Genes (COG) database. In total, we identified 5,023 core proteins and assigned dN/dS values to each ortholog in each genome. dN/dS values were binned according to clusters of orthologous genes (COGs), and t-tests were carried out by comparing the dN/dS distributions between COGs related to metabolism and defense to background genes; principally, we were interested in the COGs: “G” = carbohydrate, “U” = intracellular trafficking, secretion, and vesicular transport, “V” = defense, “I” = lipid, and “Q” = secondary metabolism.

## Results

### Sequencing and assembly of *Sympodiorosea*

We shotgun sequenced 22 strains using Illumina technology. Genome assemblies were high quality, with an average of 95% completeness under the “Ascomycota_odb10” dataset using the BUSCO (Benchmarking Universal Single-Copy Orthologs) scoring method. Average assembly size was 29.4 Mb—ranging from 26.05 to 35.8 Mb and harboring, on average, 7,724 protein-coding genes. GC% ranged considerably between 40.1% and 48.3%, with an average of 46.4% (Supplementary Table 1).

### Phylogenomics reveals a weak but significant pattern of ant-of-origin association

Pairwise average nucleotide identity (ANI) between the *Sympodiorosea* genomes ranged from 86% to 100% (Supplementary Fig. 1). Clustering ANI scores based on Euclidean distances revealed 7 distinct clusters. Owing to sometimes large nucleotide sequence differences between genomes, we elected to use protein alignments to reconstruct evolutionary relationships. We extracted 4,487 single-copy orthologs found in all 68 proteomes (22 strains from this study and 42 other fungi from 7 genera) (Supplementary Table 2). Our supermatrix alignment of 676,661 amino acids produced a maximum-likelihood phylogeny with strong support (>95%) for most nodes (Fig. 1a–c, Supplementary Fig. 2). The bootstrapped phylogeny was congruent with a phylogeny with more samples estimated using tef-1 alpha, a marker gene used in fungal phylogenetics (Supplementary Fig. 3, Supplementary Table 3). As previously demonstrated (Gotting et al. 2022; Berasategui et al. 2024), these reconstructions revealed that attine-associated antagonists are monophyletic and that *Sympodiorosea* spp. strains form a monophyletic clade within the attine associates.

**Fig. 1.**
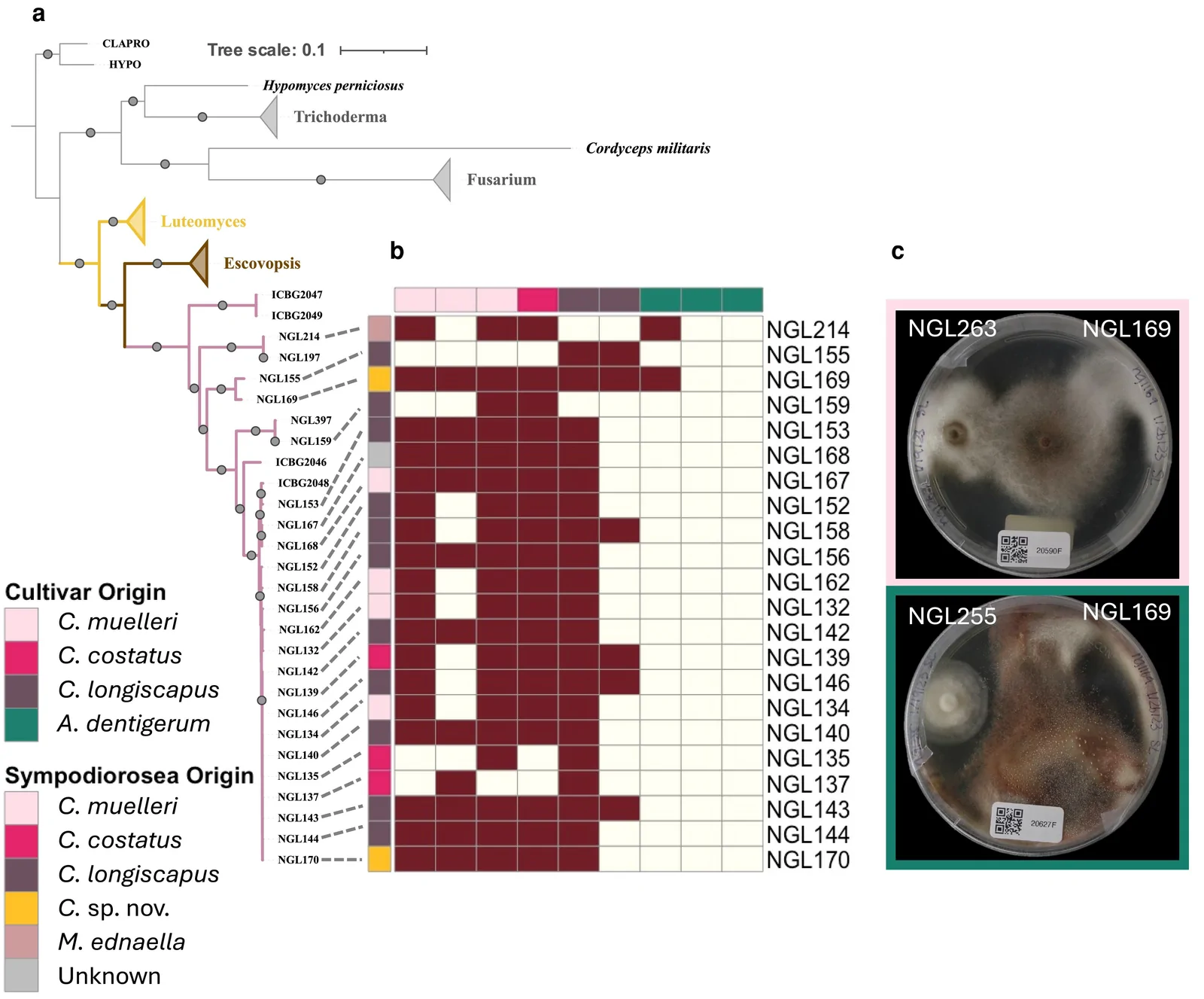
Phylogenomic placement and host use of whole-genome-sequenced *Sympodiorosea*. a) Consensus species tree, based on 4,487 shared orthologs, generated from 1,000 ultrafast bootstrap replicates. Triangles represent collapsed clades containing outgroups. Non-*Sympodiorosea*, attine-associated symbionts are represented by collapsed clades, with yellow for *Luteomyces* and brown for *Escovopsis*. *Sympodiorosea* strains are indicated on pink branches. A fully resolved phylogeny with all taxa can be found in Supplementary Fig. 1. Filled black circles indicate ultra-fast bootstrap values of >90%. The phylogeny indicates that *Sympodiorosea* spp. form a distinct clade sister to *Escovopsis* spp. isolated from higher attine and *Apterostigma* colonies. Colored boxes next to leaves indicate the ant species associated with the colony from which a sample was isolated, with numbers corresponding to associated cultivar clade (i.e. clade 1 or clade 2 in Schultz et al. 2024), when known. Within the *Sympodiorosea*, this study putatively identifies at least 6 clades that may represent distinct species of *Sympodiorosea*. b) Heatmap of host-use bioassay outcomes. Dashed lines connect position in phylogeny with row in bioassay. Filled boxes indicate that the *Sympodiorosea* strain was successful in overgrowing the cultivar, while ivory indicates that *Sympodiorosea* was inhibited by and did not successfully overgrow the cultivar. Filled boxes at the top and to the left indicate the ant-origin of the fungal isolate. Each cell represents the proportion of times a *Sympodiorosea* strain could overgrow a cultivar based on triplicate plates. c) Confrontation assays between *Sympodiorosea* strain NGL169 and a *Leucocoprinus* clade 2 cultivar (top) and NGL169 vs an outgroup cultivar (bottom).

Within *Sympodiorosea*, consistent with the ANI analysis, phylogenomic analysis supported at least 6 well-supported clades (bootstrap values >95%, Fig. 1a). Four *Sympodiorosea* strains isolated from *Myrmicocrypta* colonies, ICBG2047, ICBG2049, NGL197, and NGL214, were basal to those strains isolated from *Cyphomyrmex* colonies. The next most derived clade included NGL169 and NGL155, isolated from colonies of an undescribed *Cyphomyrmex* species and *C. longiscapus*, respectively. This clade was basal to NGL159, isolated from a *C. muelleri* colony, and NGL397, isolated from a *C. longiscapus* colony. Sister to the NGL159-NGL397 clade was a branch harboring ICBG2046, isolated from a *C. costatus* colony. ICBG2046 was the basal branch for the remaining 19 samples, of which only ICBG2048 was non-*Cyphomyrmex*-associated. Branch support was lower within the most derived clade; however, the branches containing NGL153 and ICBG2048 were highly supported (bootstrap = 100), as was an intermediate branch containing NGL168 and NGL167 (bootstrap = 100). Clustered within the largest clade were the remaining 16 *Sympodiorosea* strains: NGL152, NGL158, NGL132, NGL156, NGL140, NGL142, NGL134, NGL137, NGL146, NGL170, NGL144, NGL143, NGL135, NGL139, and a well-supported branch containing NGL162 (bootstrap = 100). We identified no clear patterns of macro-morphological diversity across the major clades when grown in vitro (Supplementary Fig. 4).

While the 7 distinct clades were not each specific to an ant species, assessment of phylogenetic signal at the strain level revealed that the ant-species-of-origin was a phylogenetically conserved trait (Pagel's *λ* = 0.842, *P* < 0.001) (Supplementary Fig. 5a). Contrastingly, we found no evidence that host cultivar clade (“clade 1” vs. “clade 2”) was correlated with relatedness between *Sympodiorosea* strains (Pagel's *λ* = 0.327, *P* > 0.05) (Supplementary Fig. 5b); notably, this trait was known for only 17 of the 22 strains, limiting power to detect an association.

### *Sympodiorosea* preferentially overgrow host similar to their native hosts

In vitro bioassays pairing *Sympodiorosea* with cultivars were performed to assess potential host range. We used cultivars isolated from *C. longiscapus*, *C. muelleri*, and *C. costatus* gardens, as well as cultivars from gardens of ants in the genus *Apterostigma dentigerum* (Supplementary Table 4). Targeting 2,121 conserved loci with custom UCE probes, we constructed a phylogenomic tree of the 9 cultivars used in this study, integrating UCEs from 99 fungal sequences, including both free-living and ant-associated cultivars (Supplementary Table 4, Supplementary Fig. 6). Bioassay results were contextualized by assigning cultivars to one of 3 clades: *Leucocoprinus* clade 1, *Leucocoprinus* clade 2, or the *Pterulaceae* clade.

As previously seen (Birnbaum and Gerardo 2016), we found *Apterostigma*-associated cultivars to be highly resistant to overgrowth by *Sympodiorosea*; *Sympodiorosea* were only able to overgrow an *Apterostigma*-associated cultivar in 2 of 69 bioassays. In contrast, the same *Sympodiorosea* were able to overgrow *Cyphomyrmex-*associated cultivars in 99 of 138 bioassays (Fig. 1b, Supplementary Fig. 7).

Within the *Cyphomyrmex* association, we identified clear patterns of overgrowth in relation to cultivar clade (Fig. 1b). *Sympodiorosea* strains isolated from *C. longiscapus* gardens overgrew *C. longiscapus* cultivars (*Leucocoprinus* clade 1 cultivars) in 59% (39/66) of bioassays. In contrast, *C. longiscapus*-derived *Sympodiorosea* overgrew *C. muelleri* and *C. costatus* cultivars (*Leucocoprinus* clade 2 cultivars) in 79% (114/144) of bioassays. *C. muelleri*-derived *Sympodiorosea* overgrew *C. muelleri* and *C. costatus* cultivars (*Leucocoprinus* clade 2 cultivars) in 81% (39/48) of bioassays but only overgrew *C. longiscapus* (*Leucocoprinus* clade 1 cultivars) in 42% (12/24) of bioassays. Interestingly, *C. costatus*-derived *Sympodiorosea* infected *C. muelleri* and *C. costatus* cultivars (*Leucocoprinus* clade 2 cultivars) in 42% (15/36) of bioassays, and overgrew *C. longiscapus* (*Leucocoprinus* clade 1 cultivars) in 50% (12/24) of bioassays. Overall, *Sympodiorosea* was significantly more likely to overgrow their native-type cultivars (i.e. cultivars from the same clade as the *Sympodiorosea*'s garden of origin) than cultivars distantly related to their native cultivar (Fisher's exact, *P* = 0.0016, Supplementary Table 5). There was a significant correlation between genetic similarity of the strains, based on ANI, and their host range, based on overgrowth patterns (Mantel test, *r* = 0.32, *P* < 0.01).

Our study also captured interactions that diverged from the norm. Strains NGL214, NGL169, NGL155, and NGL159 formed arm-like protrusions composed of mycelial mats that extended from the media, grew along the “ceiling” of Petri dishes, and finally overgrew the cultivar from the “air” (Supplementary Fig. 8a and b). This behavior was specific to interactions with the host; in the absence of hosts, these same strains failed to produce the arm-like protrusions until the plate was nearly covered, at which point protrusions would form. Even when a protrusion-forming strain was initially inhibited by the presence of a cultivar, it could deploy this growth behavior to make contact with the host while remaining distant from the presumably metabolite-laden media. This trait is specific to the basal lineages of *Sympodiorosea*, suggesting that it may have been lost in the more derived strains.

### Cazymes and proteases are highly conserved across *Sympodiorosea*

To identify variation in metabolic potential, we surveyed the distributions of gene annotations related to CAZymes, proteases, and BGCs. On average, 228 genes (range 221 to 232, median = 228) encoding CAZymes were identified in each of the 26 *Sympodiorosea* strains. CAZyme protein predictions were generally conserved across all strains (Supplementary Fig. 9a and b, Supplementary Table 6), with only one gene family, GH18, found to be significantly different in counts across the 22 *Sympodiorosea* genomes sequenced for this study (Supplementary Fig. 10). GH18 is linked to chitin degradation and was the most abundant CAZyme gene identified in our analysis. Relative proportions of CAZyme classes were conserved, with a per-genome average of 23 protein-encoding genes related to “auxiliary activities,” 5 related to “carbohydrate binding module,” 10 related to “carbohydrate esterase,” 134 related to “glycoside hydrolase,” 67 related to “glycosyl transferases,” and 5 related to “polysaccharide lyase.” From these, we surveyed the putative substrates to gain insight into the lifestyle of *Sympodiorosea*. We found glucan-degrading enzymes to be most abundant, with an average of ∼40 protein-encoding genes. Of the glucan enzyme genes, beta-glucan was the most frequently predicted substrate, with an average of 37 genes putatively related to beta-glucan degradation per genome. Glycan-degrading enzymes followed, with roughly 30 predictions per genome. An average of 15 chitinases, 14 ligninolytic enzymes, 10 xylanases, 5 arabinogalactan proteins, 5 glycogen-degrading enzymes, 5 pectinases, and 4 mannoses were predicted per genome.

There was little variation in both individual protease counts and relative abundances of protease families across the 26 *Sympodiorosea* genomes (Supplementary Table 7, Supplementary Fig. 9b). We found serine peptidases to be most common, with an average of 73 per genome, followed by glutamic peptidases with 67 genes, cysteine peptidases with 55, threonine peptidases with 19, aspartic peptidases with 16, and protease inhibitors with 3. While individual counts differed in some protein families across strains, we found no evidence that any proteases are differentially conserved within the *Sympodiorosea*.

The distribution of serine protease families is of particular interest in understanding the lifestyle of a fungus, as these hydrolytic enzymes serve critical functions in nutrient acquisition and virulence in fungi (Figueiredo et al. 2016; Li et al. 2017; Lysøe et al. 2017; Muszewska et al. 2017; Lv et al. 2023). Contractions and expansions of serine protease subfamilies have been suggested as illuminating the lifestyle and niche of a fungus (Muszewska et al. 2017). Within *Sympodiorosea*, we identified a considerable expansion of S9 (mean = 22) and S33 (mean = 21) serine peptidase subfamilies compared to other fungi, which is unexpected for pathogenic fungi in general (Muszewska et al. 2017) (Fig. 2c, Supplementary Fig. 9, Supplementary Table 8). Such patterns of expansion are most common in symbiotic fungi of plants (Muszewska et al. 2017). Furthermore, we found contractions of enzymes from the S1 (mean = 4) and S8 (mean = 3) classes and an absence of S15 enzymes, which are commonly expanded in fungal pathogens (Muszewska et al. 2017).

**Fig. 2.**
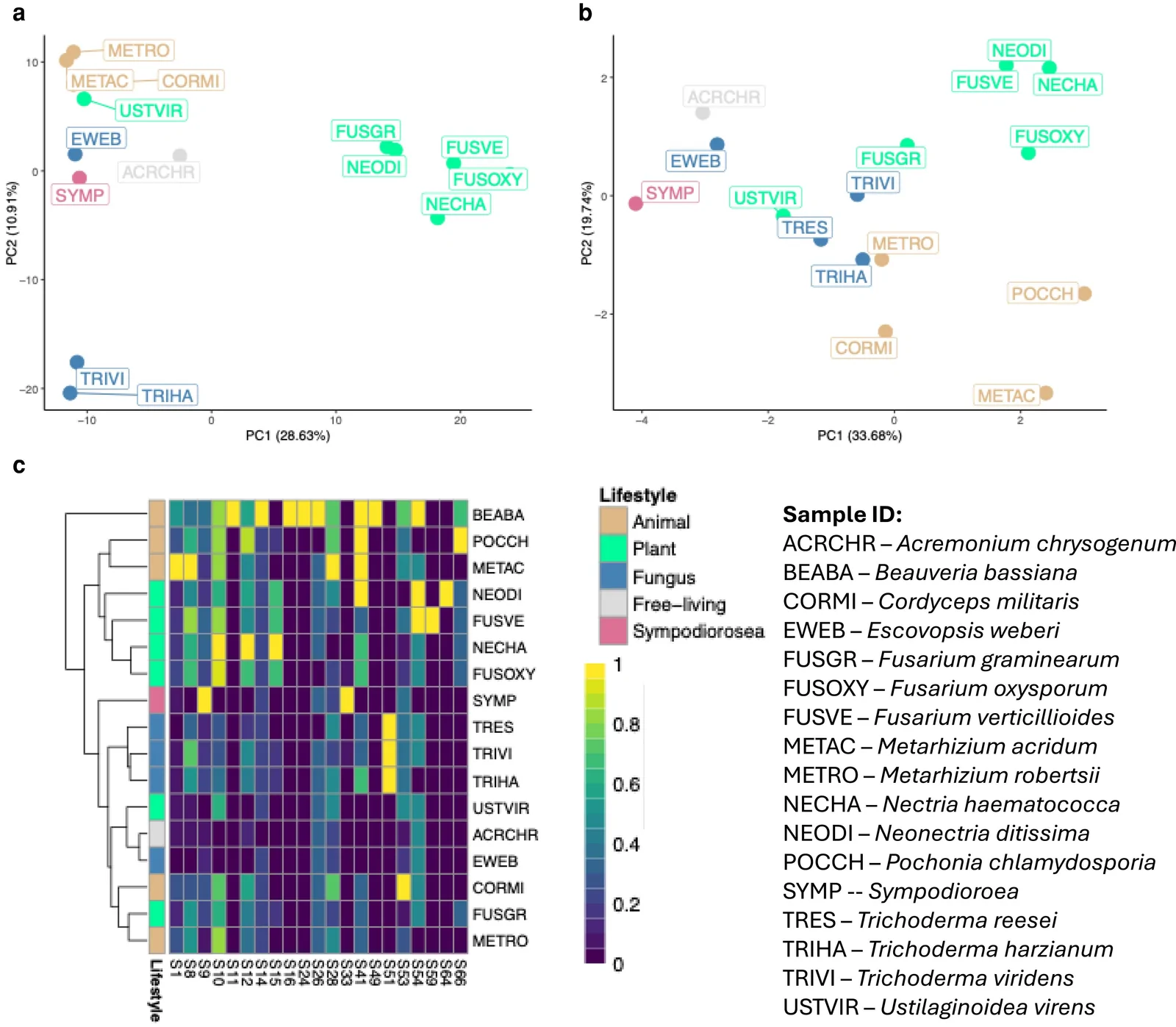
Variation in metabolic potential, focusing on genomic diversity of carbohydrate-active enzymes and proteases. Principal component analyses of Euclidean distances derived from the presence/absence of DIAMOND/Blastp protein annotations related to: a) carbohydrate-active enzymes (CAZymes) and b) serine proteases. Colors indicate lifestyle of the sample: animal pathogens = light brown, plant pathogens = light green, pathogens of fungi (mycoparasites) = blue, free-living = gray, *Sympodiorosea* = pink. c) Hierarchical clustering based on genes encoding serine proteases. Columns are normalized to the highest value to enable comparisons between protease families. Lifestyle colors follow the schema of 2a and 2b.

### *Sympodiorosea* are genomically discordant from a strictly mycoparasitic lifestyle

Fungi employ a range of proteases and CAZymes that are critical for cellular defense and nutrient acquisition, respectively. It has been suggested that patterns of contraction and expansion in these gene classes can provide insight into potential lifestyles. Of the 128 CAZymes with an assigned putative substrate, nearly 30% were specific to glucan degradation, with (37/40) enzymes specific to beta-glucan. Twenty-three percent were specific to host-glycan degradation/degradation of fungi. Glucan is among the most abundant polysaccharides in fungal tissues, indicating that *Sympodiorosea* have specialized to the most abundant sugar source in fungus-growing ant gardens. Chitin is another polymer abundant in fungal cell walls, and 12% of enzymes found in *Sympodiorosea* were specific to chitin degradation, further supporting our expectation of specialization to the most abundant nutrient source: cultivar-hyphae. Interestingly, *Sympodiorosea* house an equal number of enzymatic genes related to plant degradation (xylan and ligninolytic enzymes) as they do for chitin, a primary component of fungal cell walls.

Within the context of the order *Hypocreales*, our principal component analysis of CAZymes and serine proteases revealed contrasting results regarding how *Sympodiorosea* clusters with other *Hypocreales* fungi 4. With regard to CAZyme profiles, we found *Sympodiorosea* to be most similar to its closest relative in the dataset, *E. weberi*, which is a mycoparasite of higher attine agriculture (Reynolds and Currie 2004). Near to the attine associated fungi were the animal pathogens (2 species of *Metarhizium* and *Cordyceps militaris*) (Fig. 2a). Nearly equidistant from this cluster are 2 clusters, one containing 3 *Trichoderma* species and the other containing the *Fusarium*/*Neonectria* species. Clustering breaks down when analyzing the serine protease profiles for the same set of fungi. Notably, attine-associated fungi remain clustered; however, the mycoparasites from *Trichoderma* replace animal pathogens as the most like attine associates (Fig. 2b).

### BGC composition is indicative of phylogenetic topology

We identified 627 total BGCs grouped into 59 GCFs, with an average of roughly 22 BGCs per genome (Supplementary Table 9). We identified 59 GCFs in total, 19 of which were shared amongst at least 20 of the genomes; only 6 GCFs were unique to an individual (Fig. 3a and b). In agreement with recent studies of BGC content in the larger group containing *Escovopsis*, *Luteomyces*, and *Sympodiorosea*, we found non-ribosomal peptide synthases to be the most abundant classified BGCs, with 21 total identified. Additionally, 8 terpene, 8 PKS (7 “PKS_other,” 1 PKS Type I), and 6 hybrid BGCs were found. We identified groups of GCFs that correspond to the 3 described metabolites produced by *Escovopsis weberi* (Supplementary Table 10): melinacidin IV (Supplementary Fig. 11), shearinine D (Supplementary Fig. 12), and emodin (Supplementary Fig. 13) (Heine et al. 2018). Twenty-one BGCs were classified as “other.” We identified unique BGCs in NGL214, NGL169, NGL155, and NGL143. The assemblies of NGL155 and NGL143, however, while largely complete, were 2 of the most fragmented assemblies, and, as such, the uniqueness of these BGCs should be interpreted cautiously. To understand if genome fragmentation biases BGC predictions, we tested for correlation between the number of predicted BGCs and the number of contigs and found no relationship (*r* = 0.02).

**Fig. 3.**
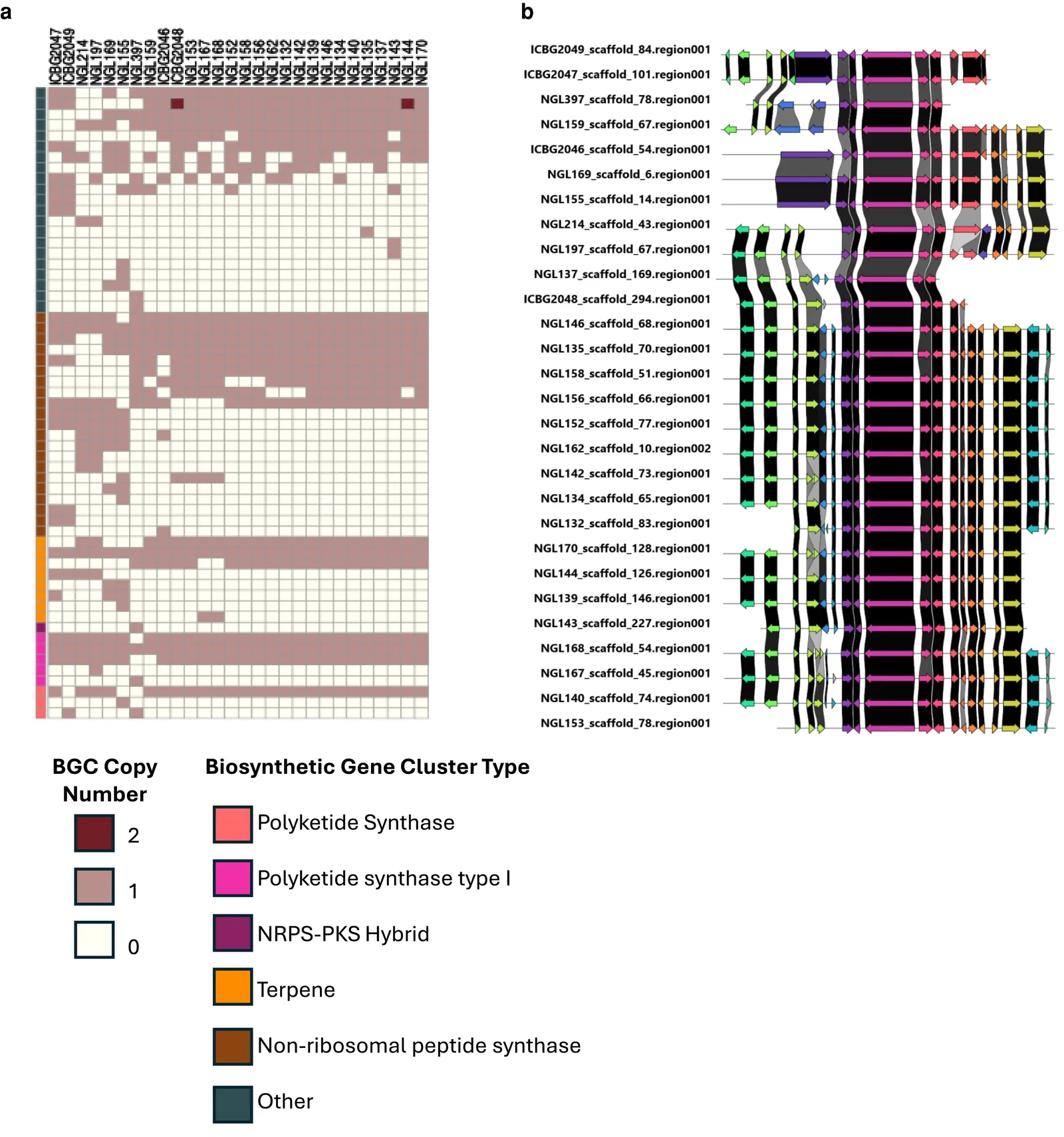
Biosynthetic gene cluster family presence and absence. a) Rows, corresponding to *Sympodiorosea* strains, are ordered as in Fig. 1a–c, and columns represent the gene cluster families (GCFs) as identified through BiG-SCAPE. Ivory-colored boxes represent the absence of a Gene Cluster Family (GCF), light brown indicates a single gene copy, and dark brown indicates a duplication of a GCF. In total, 46 GCFs were identified across the 22 focal strains in our study. Each biosynthetic gene cluster was assigned to a family based on the “Biosynthetic Gene Cluster Type” annotation column. b) Visualization of an alignment of the BGC likely encoding the compound melinacidin IV, comprising BGC families FAM00246 and FAM00381.

To test for association between phylogenetic relatedness and biosynthetic gene content, we employed Mantel tests of correlation, with the primary hypothesis that ANI is strongly associated with biosynthetic gene content. Consistent with our expectations, we found that ANI is most strongly associated with the presence/absence of BGCs (*r* = 0.84, *P* < 0.0001), followed by CAZymes (*r* = 0.80, *P* < 0.0001), and proteases (*r* = 0.66, *P* < 0.0005).

### Purifying selection structures the metabolic capacity of *Sympodiorosea*

We hypothesized that COGs involved in metabolism diversify as a result of chemical evolution in response to the hosts. Similarly, we expected metabolic related COGs to have significantly different distributions of dN/dS values compared to non-metabolic genes, which we hypothesized were more likely to exhibit signatures of purifying selection (Supplementary Table 11).

Generally, *Sympodiorosea* exhibit evidence of purifying selection (mean dN/dS = 0.134). In contrast to our expectations; however, we found that selection is generally acting to reduce diversity in metabolic-related genes relative to background genes (*P* < 0.001, Fig. 4a and b). We sought to understand which genes, if any, contributed to the selective differences between metabolic genes and background. Within the metabolism-associated orthologs, COG “Q,” related to secondary metabolism, and COGs “G,” “I,” and “E” corresponding to carbohydrate, lipid, and amino acid metabolism, harbored reduced diversity relative to other metabolic-related COGs. Mean dN/dS values were lower in “Q,” “G,” “I,” and “E” than in the remaining metabolic-COGs, which suggests that these COGs experience purifying selection relative to the genome and other non-metabolic-related genes.

**Fig. 4.**
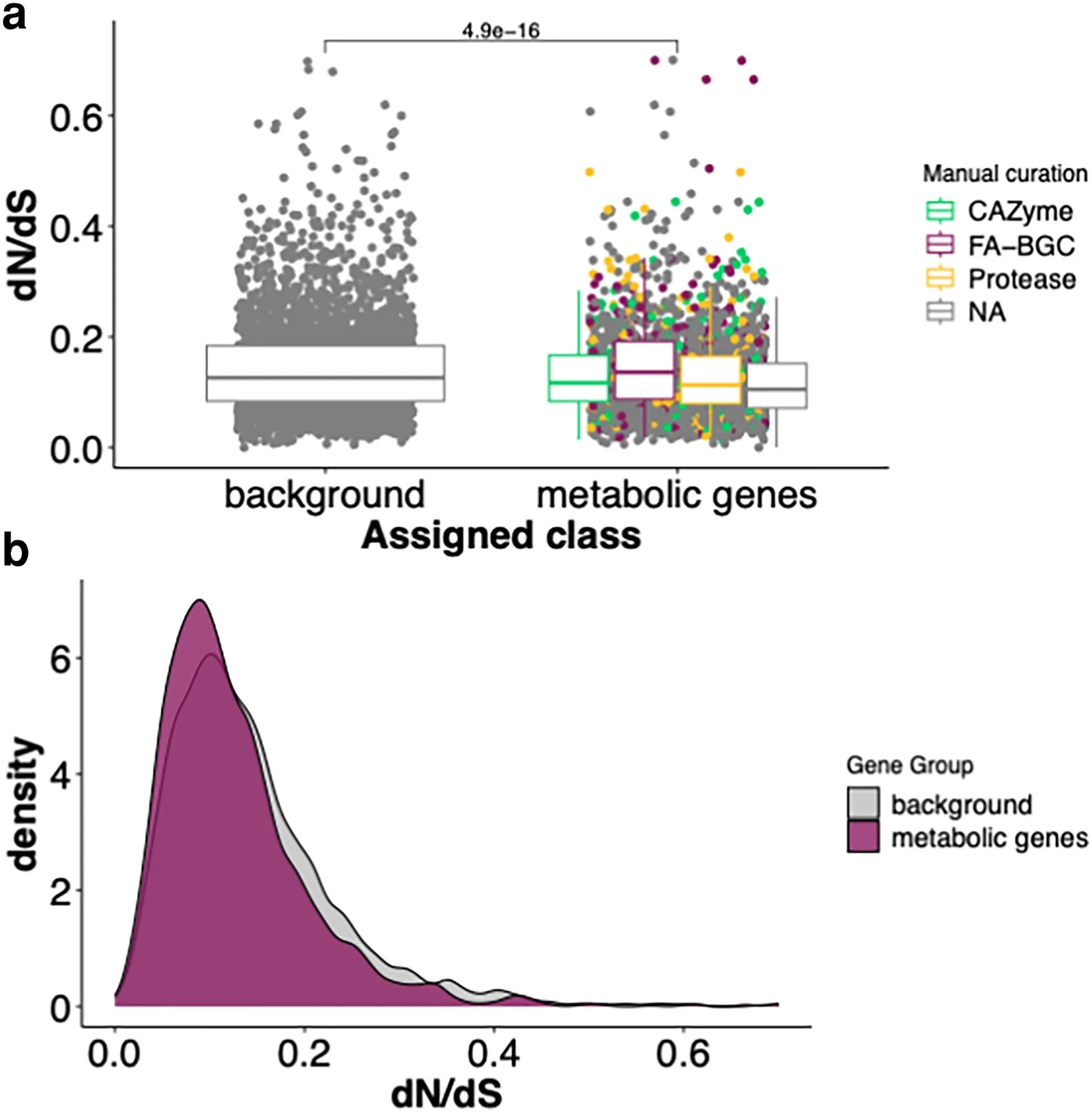
Signatures of selection. a) dN/dS values for 5,049 orthologous protein predictions. Metabolism-associated genes in gray were not assigned a gene annotation but were implicated in metabolic activities through Gene Ontology assignment. b) Density plot of dN/dS values colored by their assignment to either the background or as a putative metabolic gene.

We sought to confirm these findings by comparing selection acting on genes annotated as proteases, BGCs, and CAZymes to each other and to the rest of the genome (Fig. 4a and b). Interestingly, only the BGCs were significantly different from the background genomic content (Supplementary Fig. 14), suggesting a role of diversifying selection in BGC evolution. However, this result is driven in part by a few genes exhibiting signatures of diversifying selection; when these outliers are removed, we do not see significant evidence of diversifying selection in BGCs genes relative to the background (*P* = 0.077). The impact of outliers on the signal of selection is further highlighted by nearly identical ANI between pools of background genes and metabolism-associated genes (Supplementary Table 12), with metabolic genes only slightly more similar on average. Thirty-eight of the 47 outliers were annotated as “hypothetical proteins” without a putative function. The 9 known genes included: PEX29 (Vizeacoumar et al. 2003) responsible for peroxisome size and maintenance, IPO4 (Jäkel et al. 2002) responsible for intracellular trafficking/secretion, MIB1 (Nagase et al. 2000) involved in carbohydrate metabolism, YPD1 (Schruefer et al. 2021) involved in signal transduction, PFY1 (Ostrander and Gorman 1997) involved in actin polymerization, SUP35 (Dagkesamanskaya and Ter-Avanesyan 1991) involved in translation termination of GTPase and SQS1 (Kribii et al. 1997) involved in squalene synthesis, and a zinc finger. Additionally, we identified 19 genes putatively implicated in fungal virulence (Supplementary Table 13, Supplementary Fig. 15) whose function and roles in diversifying selection are explored further in the discussion.

## Discussion

We investigated functional, genomic, and phenotypic diversity of *Sympodiorosea* spp. to gain insight into their evolution in response to interactions within the fungus-growing ant symbiosis. Our study is the first to examine intraspecific diversity at the whole-genome-level within this group of fungi. Combined with bioassay experiments, we were able to link genomic variation in metabolism to variation in host interactions. Our study aligns with recent work at a broader taxonomic scale that also demonstrated a clear link between biosynthetic potential, symbiont lifestyle, and phylogenetic relationships (Gotting et al. 2022; Berasategui et al. 2024). These results can be used as the basis for further experiments focusing on the mechanisms driving evolution in this coevolved system.

### Bioassays reveal variation in ability to overgrow host cultivars

Host-specificity may evolve due to prolonged, repeated interactions with a host population. *Sympodiorosea* and its allies have never been isolated outside of an attine garden, suggesting a specialized lifestyle in attine agriculture. Past work has demonstrated specificity between *Sympodiorosea* and their “native hosts” or the attine species from which they were isolated (Gerardo et al. 2004, 2006; Reynolds and Currie 2004; Birnbaum and Gerardo 2016; Custodio and Rodrigues 2019). Assuming that *Sympodiorosea*, like *Escovopsis* spp., are mycoparasites (Currie et al. 1999a; Gerardo et al. 2004; Birnbaum and Gerardo 2016; Batey et al. 2020), successful interactions, from their perspective, occur when the host cultivar has been sensed, initiating chemotaxis toward the host (Gerardo et al. 2006). Then, the *Sympodiorosea* must overcome host-secretions and overgrow the cultivar biomass. For both *Escovopsis* and *Sympodiorosea*, interaction outcomes become predictably incompatible as phylogenetic distance increases for hosts (Gerardo et al. 2006; Birnbaum and Gerardo 2016). Namely, we confirm findings regarding in vitro interactions, where *Sympodiorosea* have a preference for native cultivar hosts. Additionally, we confirm previous findings (Birnbaum and Gerardo 2016) that *C. longiscapus*-associated *Sympodiorosea* overgrow a broader range of hosts than their counterparts from *C. muelleri* and *C. costatus* colonies.

### Phylogenetic relationships of *Sympodiorosea* contextualized

We sequenced 22 *Sympodiorosea* genomes and included 6 previously sequenced *Sympodiorosea* genomes in our analyses. Of these 28 strains, 22 were isolated from colonies of *Cyphomyrmex* ants. Our results identified a significant phylogenetic signal of native ant host. However, we found no significant phylogenetic signal of cultivar association (i.e. clade 1 or clade 2 cultivars). The lack of phylogenetic signal linked to the native cultivar clade could arise if only a few key genes drive evolutionary interactions with cultivars. For example, cultivar-derived selective pressures may only be detectable within specific gene sets (e.g. primary and secondary metabolism) involved in the maintenance of host interactions. In this case, coevolution between cultivars and *Sympodiorosea* might be masked by selection driven by other forces, including the ants and other microbes, or genetic drift. This, however, is not corroborated by previous research using both marker genes and genome-wide amplified length polymorphisms, which both indicated a phylogenetic signal of cultivar association (Gerardo et al. 2004; Birnbaum and Gerardo 2016). This also contrasts with bioassays, both in our study and in previous studies (Birnbaum and Gerardo 2016), which demonstrated that *Sympodiorosea* preferentially overgrow the cultivar type grown by their “native” ant species, indicating specificity. Instead, we hypothesize that small sample sizes collected over long-time scales may mask phylogenetic structure resultant from association with a particular cultivar clade, particularly if evolutionary dynamics include host switching over time.

This explanation is supported by the phylogenetic mixing of *Sympodiorosea* associated with different fungus-growing ant species. *C. muelleri* has been shown to accept the cultivar isolated from *C. longiscapus* colonies, but not vice versa (Mueller et al. 2004; Mehdiabadi et al. 2012), indicating that an opportunity exists for cultivars, and possibly *Sympodiorosea*, to be transmitted between colonies of different species of fungus-growing ant. It is therefore plausible that *Sympodiorosea* coevolves in localized “hotspots,” where it is shared between closely related species. Isolated communities may experience genetic drift, which may appear as specific coevolution within small localities but diffuse over the span of a country. Paired sampling of cultivars and antagonists from the same colonies and at regular time intervals will be necessary to understand the temporal dynamics of specialization.

### BGCs are highly conserved

Corroborating other reports, we found that genome reduction has largely eliminated BGC diversity within *Sympodiorosea* relative to other non-ant associated *Hypocreales* fungi, which harbor double the BGCs per genome, on average (Gotting et al. 2022; Berasategui et al. 2024). With an average of 22 BGCs per genome, *Sympodiorosea* have experienced a major loss in biosynthetic potential, likely resulting from a transition from a free-living to a symbiotic lifestyle. We hypothesize that the remaining BGCs are retained out of necessity for their lifestyle within fungal gardens.

In the case of the known BGCs, emodin, shearinine D, and melinacidin IV, we identified distinct clustering of corresponding GCFs between the large, derived clade and the clade that contains NGL169, NGL155, NGL214, and NGL159, which may reflect divergent selective pressures acting across the *Sympodiorosea* strains in our study. Considerably more work, however, is needed to capture the depth and breadth of the metabolome in these antagonists. Altogether, the majority of BGCs had no known analog and represent an interesting avenue of discovery. Future work should characterize the specialized metabolism of *Sympodiorosea* to understand how genomic differences in the metabolic gene repertoire are manifested. This would help to close the gap in our understanding of the mechanisms of microbial interaction in the fungus-growing ant system.

### Broad patterns of selection in *Sympodiorosea*

Genome streamlining captures the general process by which host-associated organisms lose genes related to a free-living lifestyle (Katinka et al. 2001; Moran 2002; Pombert et al. 2013; Fijarczyk et al. 2025). This process acts upon antagonistic symbionts associated with fungus-growing ant agriculture and is implicated as the main driver of genomic diversification for *Escovopsis*, *Luteomyces*, and *Sympodiorosea* (de Man et al. 2016; Gotting et al. 2022; Berasategui et al. 2024).

Genome streamlining can work synergistically with purifying selection to reduce functional diversity. Selection of this kind is common in symbionts, where they are experiencing selective pressure from both the hosts to circumvent defenses and competition with the local microbial community (Moran 2002; de Man et al. 2016; Berasategui et al. 2024; Fijarczyk et al. 2025). In line with other studies of genomic selection in pathogenic fungi, we found genomic variation in *Sympodiorosea* is constrained by purifying selection (Silva et al. 2015; Kobmoo et al. 2018). Combined with the nearly identical ANI between strains, our genomes are consistent with patterns of reduced genomic diversity resultant from a symbiotic lifestyle.

With their evolutionary success largely predicated on streamlining essential processes, symbiont genomes can diversify at “hotspots” of evolution, where nonsynonymous mutations accumulate; these genomic regions often contain genes implicated in virulence and defense. We identified a significant difference in selection between the “background” genomic content and metabolic genes, including BGCs, CAZymes, proteases, and lipases. dN/dS values derived from BGCs were also significantly different than either CAZymes or proteases, suggesting that the *Sympodiorosea* genome evolves in a multispeed or multi-compartment system (Barelli et al. 2016; Frantzeskakis et al. 2018). Differences between BGCs and other gene groups may indicate that BGCs undergo diversifying selection, leading to the maintenance of functional diversity. Ultimately, our findings are consistent with the hypothesis that changes in BGCs are a principal driver of evolution in *Sympodiorosea*.

### Genes with elevated dN/dS

dN/dS captures the ratio of nonsynonymous vs synonymous mutations that accrue in a particular gene. This measure assumes that synonymous mutations in a protein-coding gene will produce a functionally similar protein, whereas nonsynonymous mutations are expected to produce variation that selection can act upon. Nonsynonymous mutations are often deleterious to the organism. Subsequently, elevated dN/dS indicates an advantage resulting from diversifying selection, which is pertinent to the evolution of novel defenses, virulence factors, and for escaping host recognition. Three genes with elevated dN/dS were components of BGCs identified by antiSMASH. Peroxisomes are functionally diverse in filamentous fungi and are often implicated in the production of antibiotics and other secondary metabolites (van der Klei and Veenhuis 2013).

Ortho5377, another gene with elevated dN/dS, likely encodes a telomeric enzyme. In fungi, BGCs are often located in recombinant, sub-telomeric regions of chromosomes where high-repeat nucleotide content and transposable elements often reside. In these regions, genomic stability is lower, and, subsequently, the likelihood of BGCs acquiring variation is higher (Palmer and Keller 2010; Zhang et al. 2024). Altered telomeric enzymes present an interesting path of evolution for *Sympodiorosea*, particularly as these enzymes may provide an evolutionary “playground” for beneficial mutations to accrue in functionally important regions of the genome.

F-box proteins were also implicated as drivers of genomic diversity in *Sympodiorosea*. These proteins function as major regulators of basic cellular functions as well as highly specific activities, like host-sensing and nutrient acquisition (Jonkers and Rep 2009; Liu and Xue 2011; Karahoda et al. 2022). These proteins are often modulated and degraded with ubiquitin-related metabolism, suggesting that transcriptional differences are important in differentiating these fungi from one another. It is thus interesting that we identified multiple instances of F-box protein-encoding genes exhibiting diversifying selection, which may reflect evolutionary pressure imposed by cultivar presence and chemistry. Also potentially related to cultivar-induced selection, 2 orthologs of interest likely encode functions related to carbohydrate metabolism. Orth5698 is a CAZyme from the GH3 family primarily responsible for the degradation of cellobiose into glucose. As *Cyphomyrmex* spp. and other lower attine ants provision their gardens with organic detritus, it is possible that these fungi genetically track the most abundant carbon sources in the garden.

Lastly, an extracellular metalloprotease, Ortho5813, was also under elevated rates of selection. Metalloproteases contribute essential functions related to degradation of host tissues, nutrient acquisition, and evasion of host defenses. Whether these functions are pertinent in relation to interactions with the cultivars, the ants, or other members of the garden community remains an interesting path of investigation, particularly as it pertains to the principal drivers of evolution for *Sympodiorosea*.

### A broader lifestyle than presumed?

*Sympodiorosea* have long been considered parasites that specialize in attacking and consuming the fungal cultivars grown by lower fungus-growing ant species (Gerardo et al. 2004, 2006; Montoya et al. 2021). We found signatures within the genome consistent with this perspective but also some evidence that *Sympodiorosea* may consume non-fungal tissues. Specifically, in assessing putative substrates of CAZymes encoded in the sequenced genomes, we found strong evidence for utilization of the most abundant polysaccharides in fungi, but we also found evidence for utilization of lignin, starch, and xylem, which are specific to plants. Lignin and xylem are recalcitrant, plant-specific polymers critical to cellular structure and nutrient transport. The presence of these enzymes-encoding genes may result from a more complex lifestyle in association with plants, or they may serve an ecological role in the garden by degrading carbohydrates that are otherwise inaccessible. Importantly, when we assessed CAZyme profiles within the context of putative lifestyles, we found that clustering is likely shaped by shared evolutionary history, indicating that these genes may evolve neutrally or through purifying selection.

It is plausible that *Sympodiorosea* retain the ability to degrade plant matter as a remnant of a past free-living or endophytic lifestyle, or that they retain the metabolic capacity to degrade the byproducts produced by cultivars as they degrade plant substrates. This is similar to the parasitic fungus *Ophiocordyceps australis*. These fungi live a “double-life,” where the fungus can activate specific enzymes in response to ant or plant interactions (de Menezes et al. 2023). While *Sympodiorosea* have never been found to infect plants, it is possible that their introduction to ant gardens coincides with the introduction of fresh material to the garden. It is also plausible that *Sympodiorosea* are tracking the most abundant nutrients in the garden, requiring some degradation of foraged plant matter. With an abundance of fungal derived carbohydrates available, we find the former scenario to be most plausible. Future work should experimentally validate the ability of *Sympodiorosea* to utilize alternative carbohydrates.

Lastly, while CAZyme diversity tracks phylogenetic diversity, we found much weaker clustering when assessing the serine protease profiles across *Hypocreales* fungi, suggesting that these genes may be important for lifestyle specialization. In this context, *Sympodiorosea* were most similar to *E. weberi*, a specialized mycoparasite of higher attine agriculture, and the free-living saprotroph *Acremonium chrysogenum*. Mycoparasites from *Trichoderma* and plant pathogens from *Ustilaginoidea virens* and *Fusarium graminearum* comprised the nearest cluster to that containing *Sympodiorosea*. Discordance between CAZyme and serine protease genetic variation invites future study of how serine proteases contribute to functional variation in *Sympodiorosea* and the family *Hypocreales* more broadly.

## Conclusion

We sought to understand how selection shapes genomic diversity related to secondary metabolism, with the goal of elucidating potential mechanisms of evolution of these fungi in response to association with the fungus-growing ant symbiosis. Our analysis revealed considerable phenotypic diversity derived from modest genomic diversity in a small pool of samples. *Sympodiorosea* genomic evolution appears to be largely shaped by purifying selection. Exceptions include select genes involved in virulence and membrane protein synthesis, telomeric modification, and F-box proteins. Relative to the genome, these genes exhibit diversifying selection, indicating potential pathways for these fungi to specialize to the agricultural lineage of their origin, or perhaps to some other biotic factor to which we are not aware. Combined with our results demonstrating that the presence/absence of BGCs serves as a phylogenetic trait, we posit that gain/loss of metabolic genes serves as a putative mechanism for genomic diversification and, subsequently, coevolution between antagonists and the fungal gardens.

Future research should explore the mechanistic basis of evolution through functional characterization of genes of interest and eventual screening of mutants against cultivars to identify altered biological processes. Additionally, the identification of metabolites that modulate interactions between cultivars and *Sympodiorosea* could provide a considerable step towards uncovering the mechanistic basis of fungal evolution in the fungus-growing ant system.

## Supplementary Material

jkag251_Supplementary_Data

## Data Availability

Fungal strains are available upon request. File “Supplementary Methods and Figures” contains detailed descriptions of reagents not included in the main text. File “Supplementary Tables” contains sample metadata, assembly statistics, and relevant data for comparative genomics. Sequence data are available at GenBank under Bioproject PRJNA1254262, and the accession numbers are listed in file “Supplementary Tables 1 and 2.” Supplemental material available at G3 online.
