# Supplementary material for: Comparative genomics of Sympodiorosea identifies genome evolution mediated through selective pressure on the metabolic gene repertoire
Source: G3 (Bethesda). 2026 Sep 21;16(11):jkag251. doi: 10.1093/g3journal/jkag251 (PMC13732433; doi:10.1093/g3journal/jkag251)
Supplement: Supplementary file 1 [file jkag251_supplementary_data.zip › Supplemental_Methods_and_Figures_G3-2026-407004.pdf]

Supplementary Methods and Figures

Comparative genomics of *Sympodiorosea* identifies genome evolution mediated through selective pressure on the metabolic gene repretoire

Supplementary Methods – Lysis Buffer Recipe

Reagent	Quantity
NaCl	150mM
EDTA pH 8.0	1 mM
Tris Base pH 8.0	10 mM
Polyvinylpolypyrrolidone (PVPP)	2%
IGEPAL-CA630	0.50%
Sodium deoxycholate	0.50%
SDS	1%
RNAse-A	100 ¬µg/mL

Fig. S1. Average nucleotide identity of the 22 strains of *Sympodiorosea* sequenced in this study.

	NGL214	NGL169	NGL155	NGL159	NGL152	NGL158	NGL132	NGL156	NGL140	NGL142	NGL134	NGL137	NGL146	NGL200	NGL170	NGL144	NGL143	NGL135	NGL139	NGL162	NGL168	NGL167	NGL153
NGL214	1.00	0.86	0.86	0.86	0.87	0.87	0.87	0.87	0.87	0.87	0.87	0.87	0.88	0.88	0.87	0.87	0.88	0.88	0.88	0.87	0.87	0.87	0.87
NGL169	0.86	1.00	0.92	0.86	0.89	0.89	0.89	0.89	0.89	0.89	0.89	0.89	0.89	0.89	0.89	0.89	0.90	0.89	0.89	0.89	0.89	0.88	0.89
NGL155	0.86	0.92	1.00	0.86	0.88	0.88	0.88	0.88	0.88	0.88	0.88	0.88	0.88	0.88	0.88	0.88	0.89	0.88	0.88	0.88	0.88	0.88	0.88
NGL159	0.86	0.86	0.86	1.00	0.88	0.88	0.88	0.88	0.88	0.88	0.88	0.88	0.88	0.88	0.88	0.88	0.89	0.88	0.88	0.88	0.88	0.88	0.88
NGL152	0.87	0.89	0.88	0.88	1.00	0.98	0.98	0.98	0.98	0.98	0.98	0.98	0.98	0.98	0.98	0.98	0.98	0.98	0.98	0.98	0.96	0.96	0.96
NGL158	0.87	0.89	0.88	0.88	0.98	1.00	0.99	0.99	0.99	0.99	0.99	0.99	0.99	0.99	0.99	0.99	0.99	0.99	0.99	0.98	0.96	0.96	0.96
NGL132	0.87	0.89	0.88	0.88	0.98	0.99	1.00	0.99	0.99	0.99	0.99	0.99	0.99	0.99	0.99	0.99	0.99	0.99	0.99	0.98	0.96	0.96	0.96
NGL156	0.87	0.89	0.88	0.88	0.98	0.99	0.99	1.00	0.99	0.99	0.99	0.99	0.99	0.99	0.99	0.99	0.99	0.99	0.99	0.98	0.96	0.96	0.96
NGL140	0.87	0.89	0.88	0.88	0.98	0.99	0.99	0.99	1.00	1.00	1.00	1.00	1.00	1.00	1.00	1.00	1.00	1.00	1.00	0.98	0.96	0.96	0.96
NGL142	0.87	0.89	0.88	0.88	0.98	0.99	0.99	0.99	1.00	1.00	1.00	1.00	1.00	1.00	1.00	1.00	1.00	1.00	1.00	0.98	0.96	0.96	0.96
NGL134	0.87	0.89	0.88	0.88	0.98	0.99	0.99	0.99	1.00	1.00	1.00	1.00	1.00	1.00	1.00	1.00	1.00	1.00	1.00	0.98	0.96	0.96	0.96
NGL137	0.87	0.89	0.88	0.88	0.98	0.99	0.99	0.99	1.00	1.00	1.00	1.00	1.00	1.00	1.00	1.00	1.00	1.00	1.00	0.98	0.96	0.96	0.96
NGL146	0.88	0.89	0.88	0.88	0.98	0.99	0.99	0.99	1.00	1.00	1.00	1.00	1.00	1.00	1.00	1.00	1.00	1.00	1.00	0.98	0.96	0.96	0.96
NGL170	0.87	0.89	0.88	0.88	0.98	0.99	0.99	0.99	1.00	1.00	1.00	1.00	1.00	1.00	1.00	1.00	1.00	1.00	1.00	0.98	0.96	0.96	0.96
NGL144	0.87	0.89	0.88	0.88	0.98	0.99	0.99	0.99	1.00	1.00	1.00	1.00	1.00	1.00	1.00	1.00	1.00	1.00	1.00	0.98	0.96	0.96	0.96
NGL143	0.88	0.90	0.89	0.89	0.98	0.99	0.99	0.99	1.00	1.00	1.00	1.00	1.00	1.00	1.00	1.00	1.00	1.00	1.00	0.98	0.96	0.96	0.96
NGL135	0.88	0.89	0.88	0.88	0.98	0.99	0.99	0.99	1.00	1.00	1.00	1.00	1.00	1.00	1.00	1.00	1.00	1.00	1.00	0.98	0.96	0.96	0.96
NGL139	0.88	0.89	0.88	0.88	0.98	0.99	0.99	0.99	1.00	1.00	1.00	1.00	1.00	1.00	1.00	1.00	1.00	1.00	1.00	0.98	0.96	0.96	0.96
NGL162	0.87	0.89	0.88	0.88	0.98	0.98	0.98	0.98	0.98	0.98	0.98	0.98	0.98	0.98	0.98	0.98	0.98	0.98	0.98	1.00	0.96	0.96	0.96
NGL168	0.87	0.89	0.88	0.88	0.96	0.96	0.96	0.96	0.96	0.96	0.96	0.96	0.96	0.96	0.96	0.96	0.96	0.96	0.96	0.96	1.00	1.00	0.98
NGL167	0.87	0.88	0.88	0.88	0.96	0.96	0.96	0.96	0.96	0.96	0.96	0.96	0.96	0.96	0.96	0.96	0.96	0.96	0.96	0.96	1.00	1.00	0.98
NGL153	0.87	0.89	0.88	0.88	0.96	0.96	0.96	0.96	0.96	0.96	0.96	0.96	0.96	0.96	0.96	0.96	0.96	0.96	0.96	0.96	0.98	0.98	1.00

1
0.98
0.96
0.94
0.92
0.9
0.88
0.86

Fig. S2. Whole-genome based phylogeny, similar to Fig . 1 but with all clades expanded to include the sample ID corresponding to entries in Supplementary Table 2. Pink indicates *Sympodiorosea* spp., brown indicates *Escovopsis* spp., yellow indicates *Luteomyces* spp., and gray indicates outgroups.

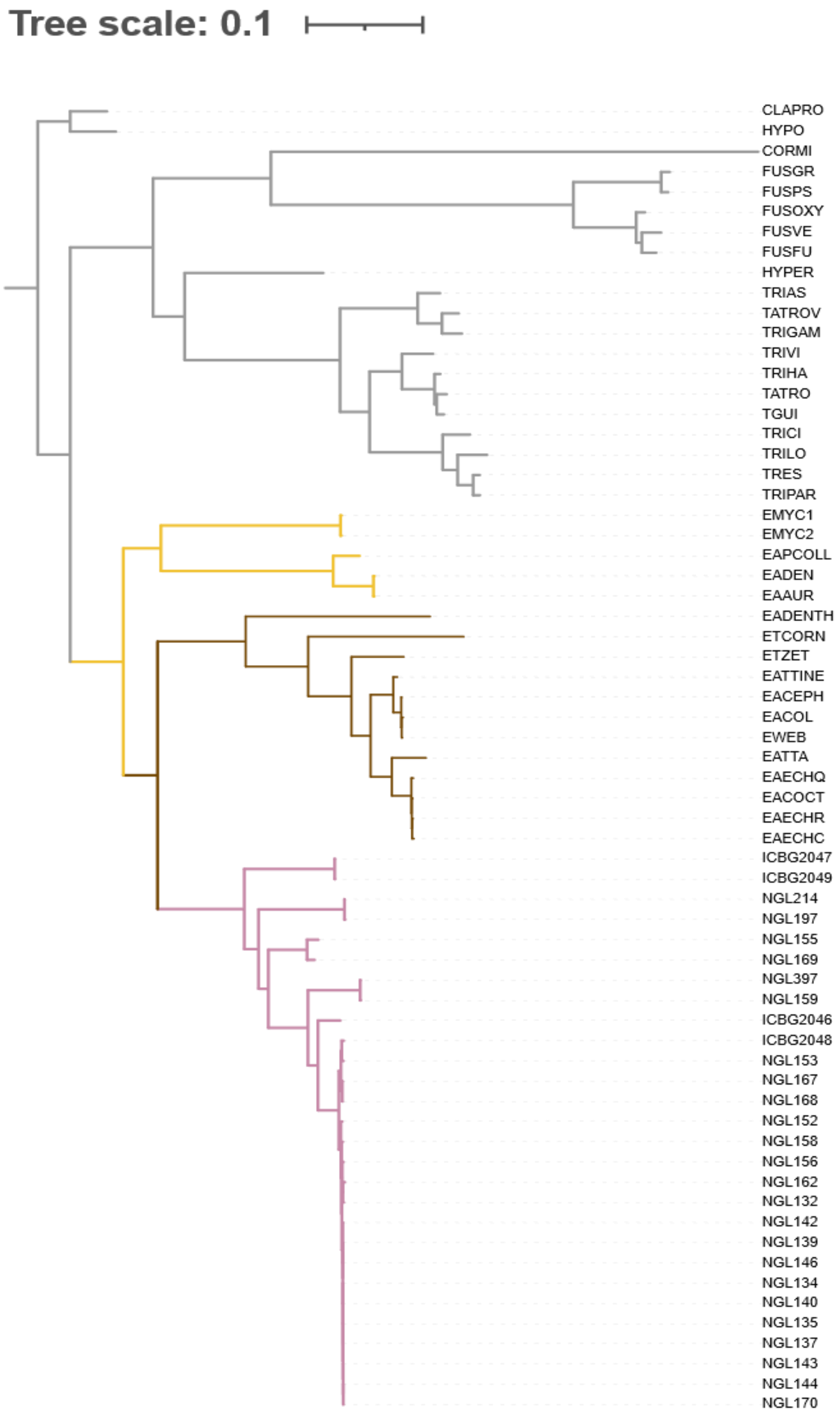

Fig. S3. Tef1-alpha phylogeny of *Sympodiorosea* included in this study and representative other strains. Outer blue bars indicate strains sequenced as part of this study. Other *Sympodiorosea* represent most other *Sympodiorosea* sequences available in NCBI. Tree visualized with iTOL.

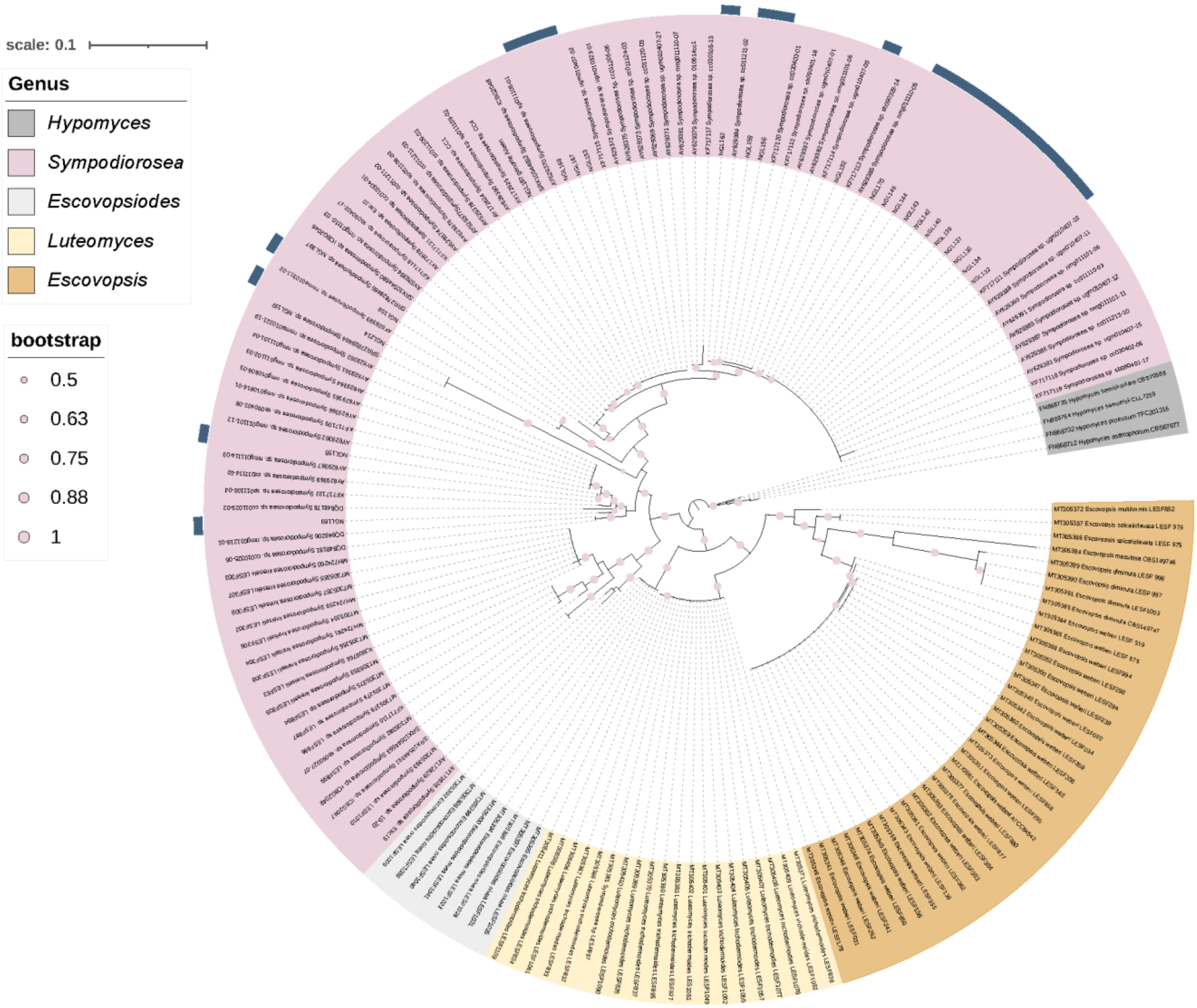

Fig. S4. Morphological Diversity of Sympodiorosea. Images were taken of culture plates after seven days of growth on potato dextrose agar. Care was taken to plate single inocula without dispersing spores across the media. Germination rates and conidia production varied between samples. Samples are ordered as in the phylogeny in Fig. 1.

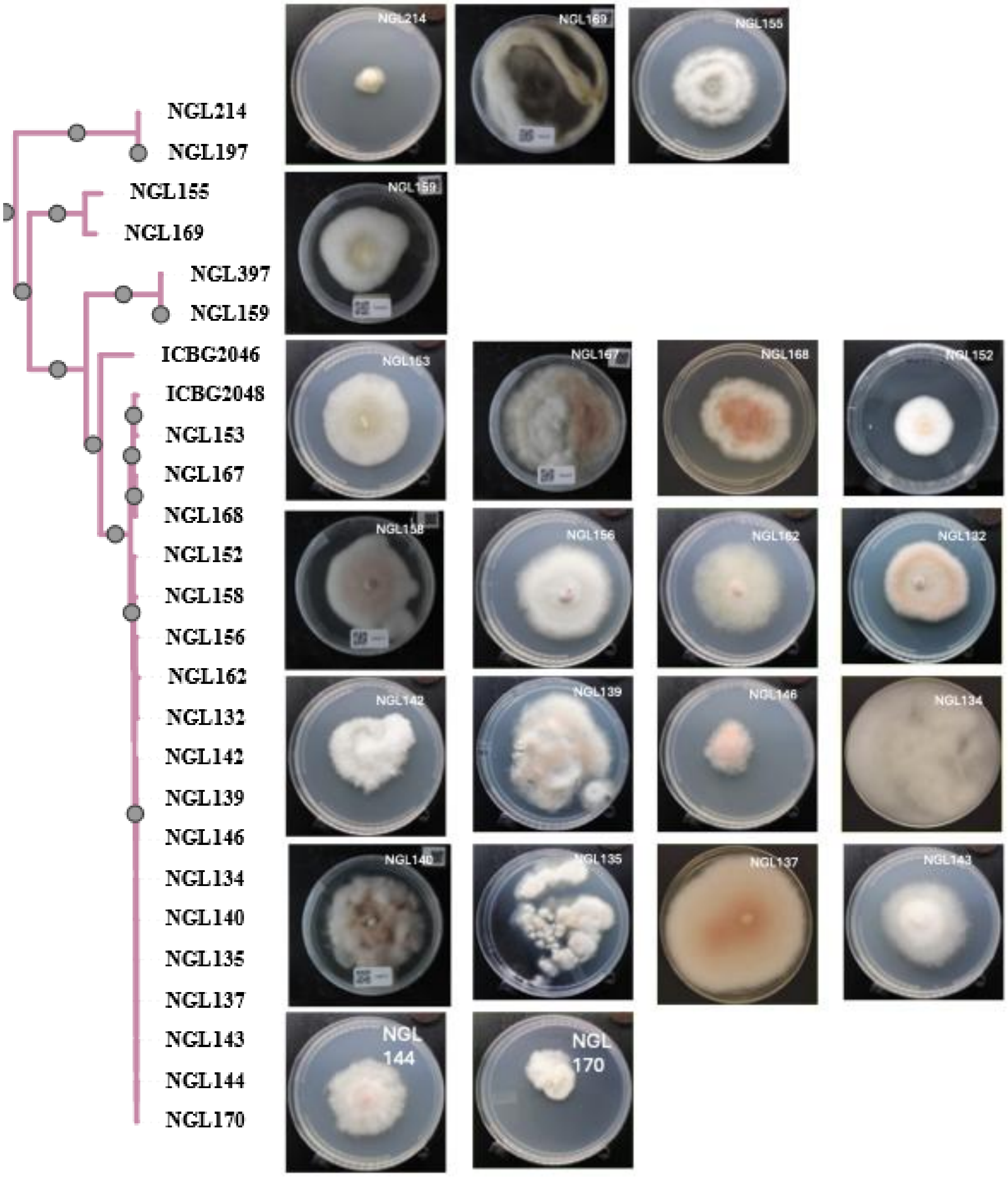

Fig. S5. Phylograms depicting associations of the *Sympodiorosea* phylogeny with A) native attine ant host and B) native cultivar clade. The position of dots on the horizontal axis indicates the ant species of origin (A) and the associated cultivar clade (B). Pagel's lambda was used as the test statistic. Corresponding test statistics and significance values reside below each phylogram. Readers should note the absence of samples NGL132, and NGL168 in **A** because these samples come from unknown attine species. NGL132, NGL168, NGL169, NGL170, NGL214, ICBG2047, ICBG2048, ICBG2049 were excluded from **B** due to absence of metadata regarding cultivar phylogenetic placement.

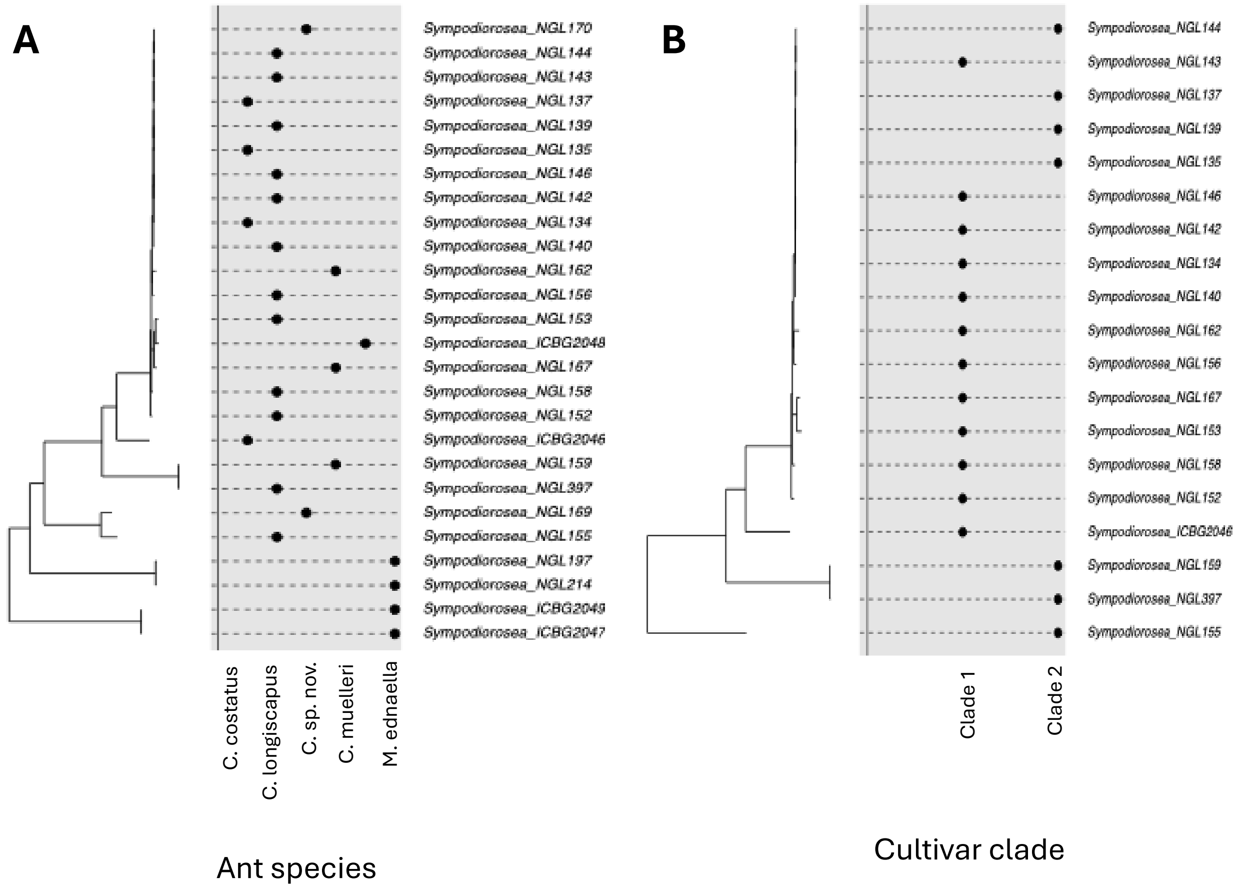

Pagel's lambda = 0.867
$p < 0.001$

Pagel's lambda = 0.327
$p = 0.851$

Fig. S6 Phylogenetic tree of cultivars used in bioassays utilizing ultra conserved elements (UCEs). Cultivars used in this study are indicated blue text. Cultivars broadly fall into two known clades, *Leucocoprinus* Clade 1, and *Leucocoprinus* Clade 2 as has been previously described (Schultz et. al 2024). As expected, outgroup cultivars isolated from gardens of *Apterostigma dentigerum* attines (NGL292, NGL255, NGL347) fall with the Pterulaceae cultivars indicated by in the green-background clades. Background color delineates primary cultivar clades.

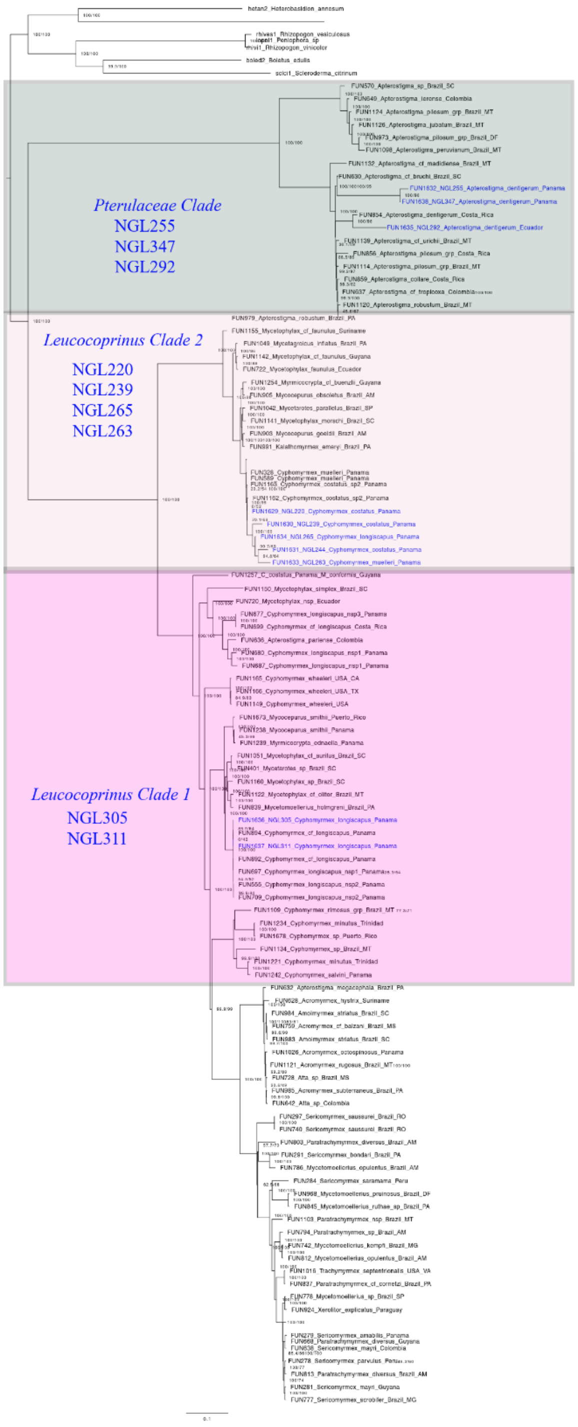

Fig. S7. Mosaic plot of bioassay outcomes constructed from contingency table (supplementary table S5) to test for links between the ability of *Sympodiorosea* to overgrow cultivars and the cultivar clade they are expected to associate with in nature.

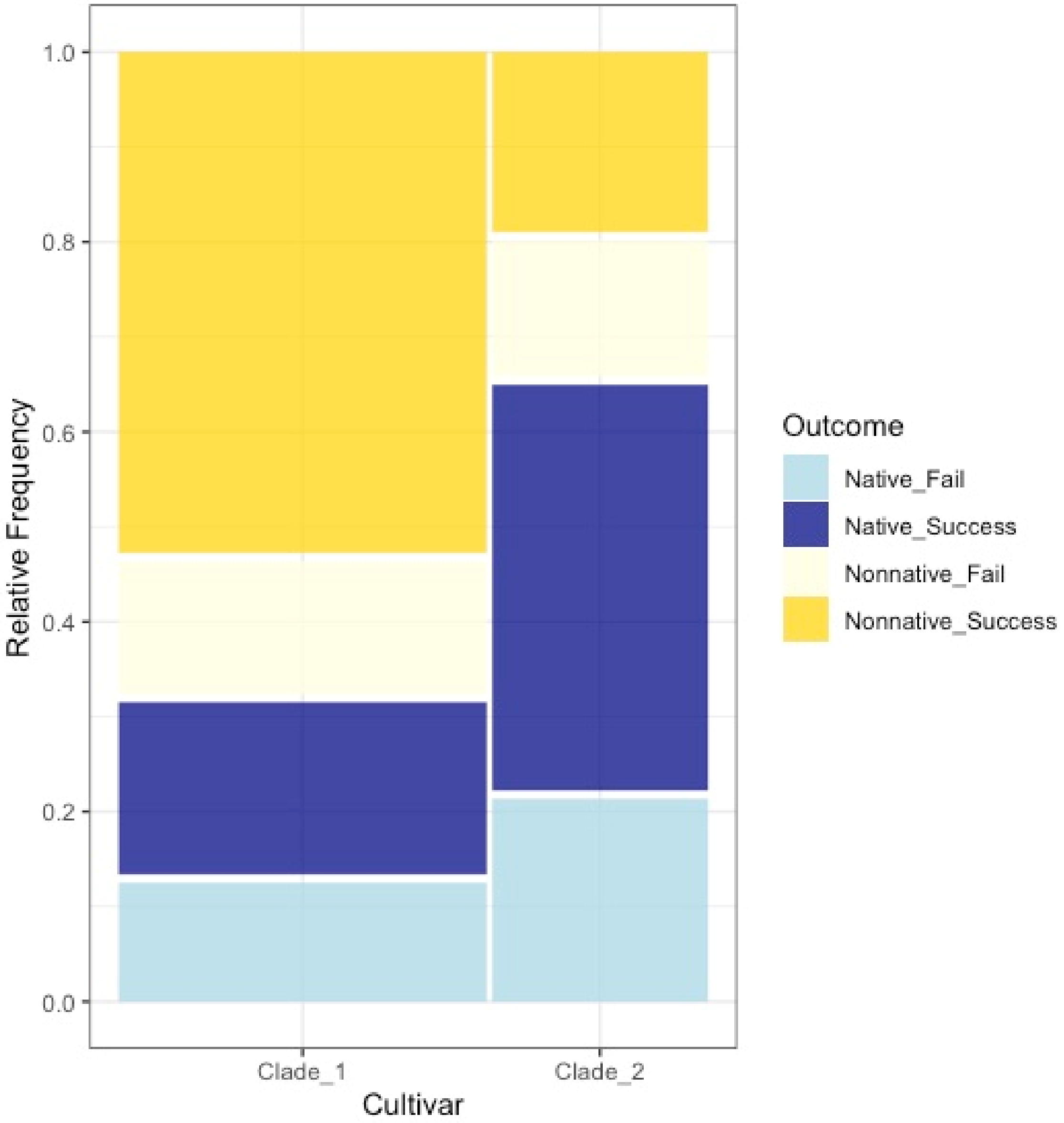

Fig. S8. A) Scoring rubric for *Sympodiorosea*-cultivar interactions. Top row depicts interactions classified as ‘no overgrowth’ with the bottom row depicting ‘overgrowth’. B) Example of NGL169 deploying ‘arm-like protrusions that rise from the agar surface to make contact with cultivar biomass. Photos span 14 days, at which NGL169 overgrows the plated cultivar.

A

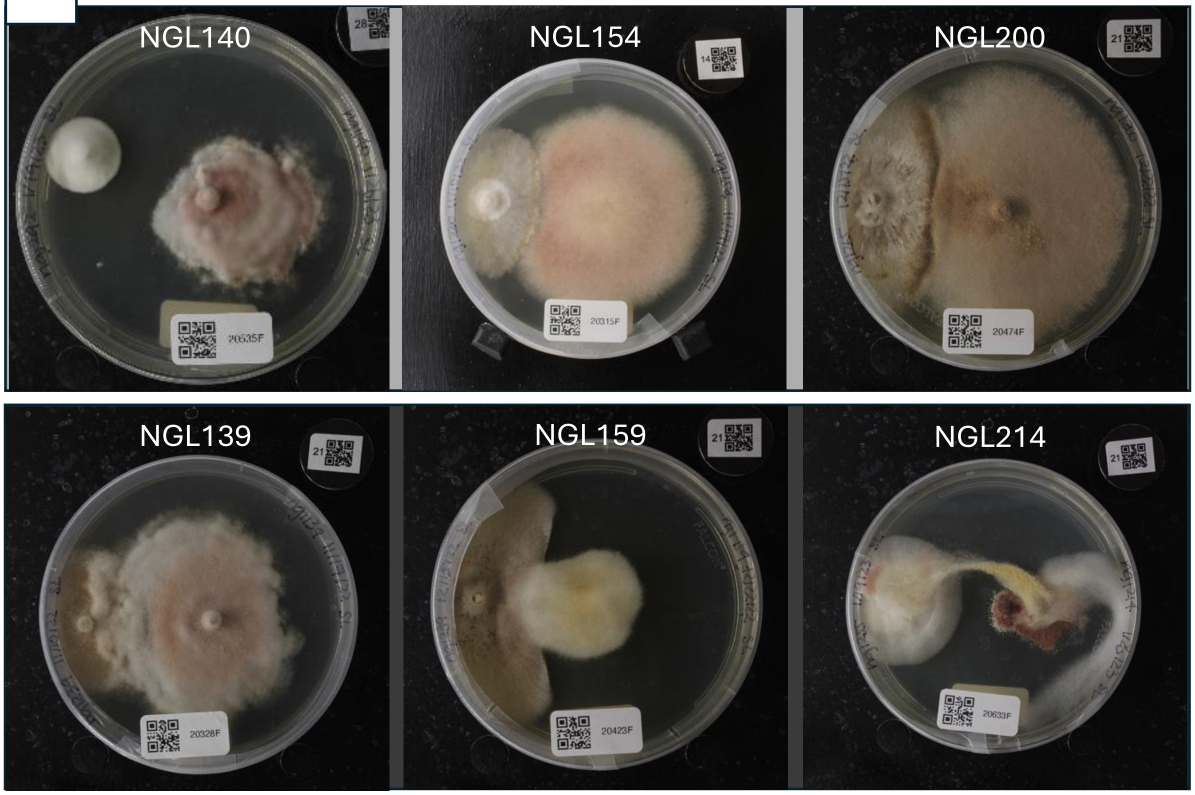

B

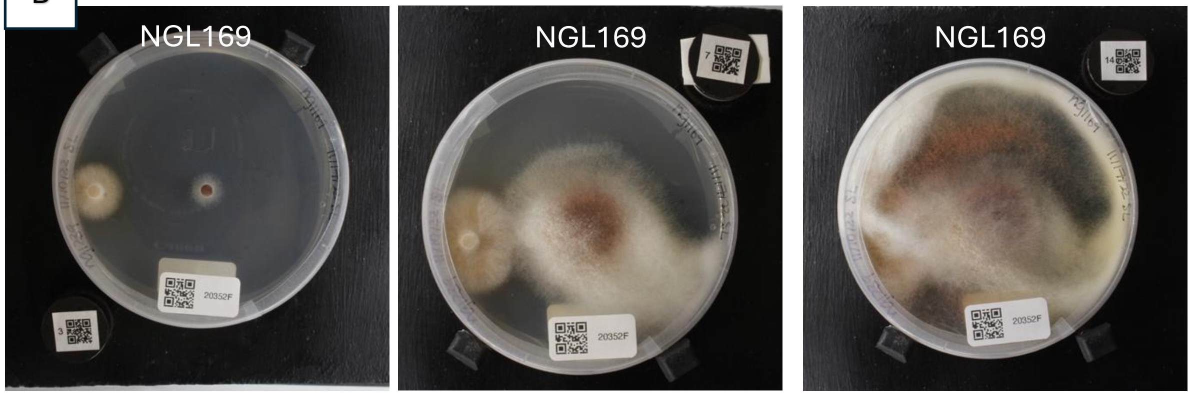

Fig. S9. Variation in metabolic potential, focusing on genomic diversity of carbohydrate-active enzymes and proteases. Heatmaps drawn based on counts of protein annotations related to: A) carbohydrate active enzymes (CAZymes) and B) proteases. In both A and B, rows are ordered according to phylogeny in Fig. 1. Darker colors indicate higher counts in both graphs, though readers should note that the shading represents different scales for A and B. For CAZymes expected to degrade extracellular substrates, the top bar indicates a putative source of the substrate (*e.g.,* the organism), and the lower bar indicates the putative substrate.

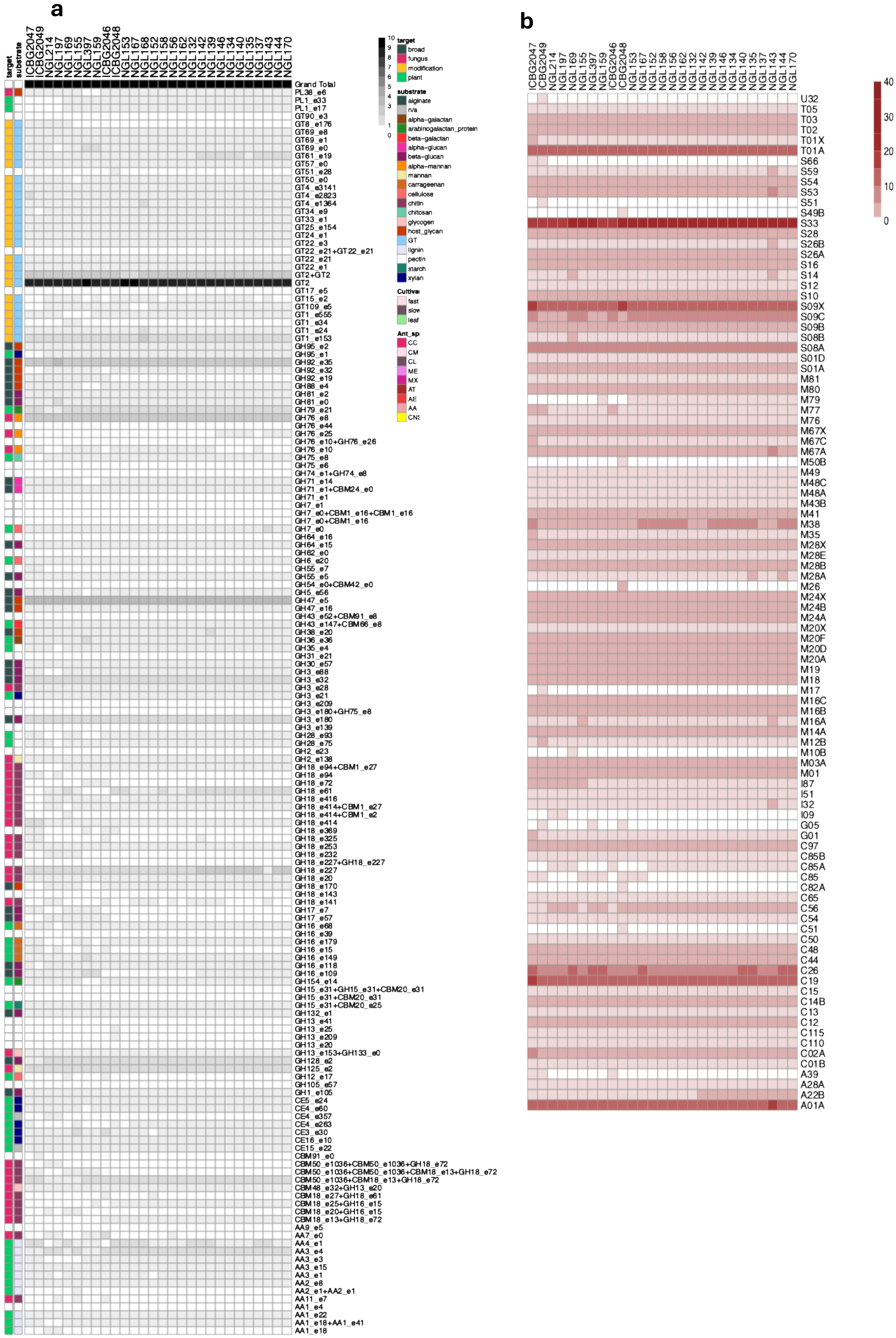
a
b
target
substrate
ICBG2047
ICBG2049
NGL214
NGL197
NGL169
NGL155
NGL397
NGL159
ICBG2046
ICBG2048
NGL153
NGL167
NGL168
NGL152
NGL158
NGL156
NGL162
NGL132
NGL142
NGL139
NGL146
NGL134
NGL140
NGL135
NGL137
NGL143
NGL144
NGL170
Grand Total
target
broad
fungus
modification
plant
substrate
alginate
n/a
alpha−galactan
arabinogalactan_protein
beta−galactan
alpha−glucan
beta−glucan
alpha−mannan
mannan
carrageenan
cellulose
chitin
chitosan
glycogen
host_glycan
GT
lignin
pectin
starch
xylan
fast
slow
leaf
CC
CM
CL
ME
MX
AT
AE
AA
40
30
20
10
0

Fig. S10. Significantly different counts of Glycoside Hydrolase 18 (GH18) genes across *Sympodiorosea*. GH18 was the only CAZyme found to be differentially conserved across *Sympodiorosea*. Members of GH18 are putative CAZyme implicated as chitinases, with additional functions as xylanase inhibitors.

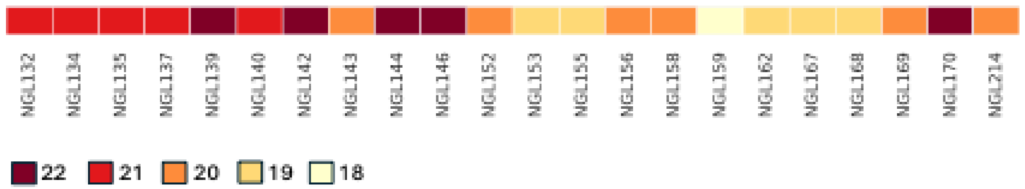

Fig. S11. Melinacidin IV analog in *Sympodiorosesa*. Alignment and visualization of the melinacidin IV analog identified from the 28 *Sympodiorosea* genomes sequenced as part of this study. Changes in the BGC gene content is reflective of the predicted phylogenomic topology, suggesting a putative mechanism for specialization to different ant agricultural regimes. Dotted lines indicate that the BGC was located on the edge of a contig, thus 'missing' genetic elements may exist in those genomes where the search algorithm could not locate them adjacent to the major BGC motifs. Melinacidin IV was first characterized in the sister genus *Escovopsis,* which is obligately associated with higher attine agriculture.

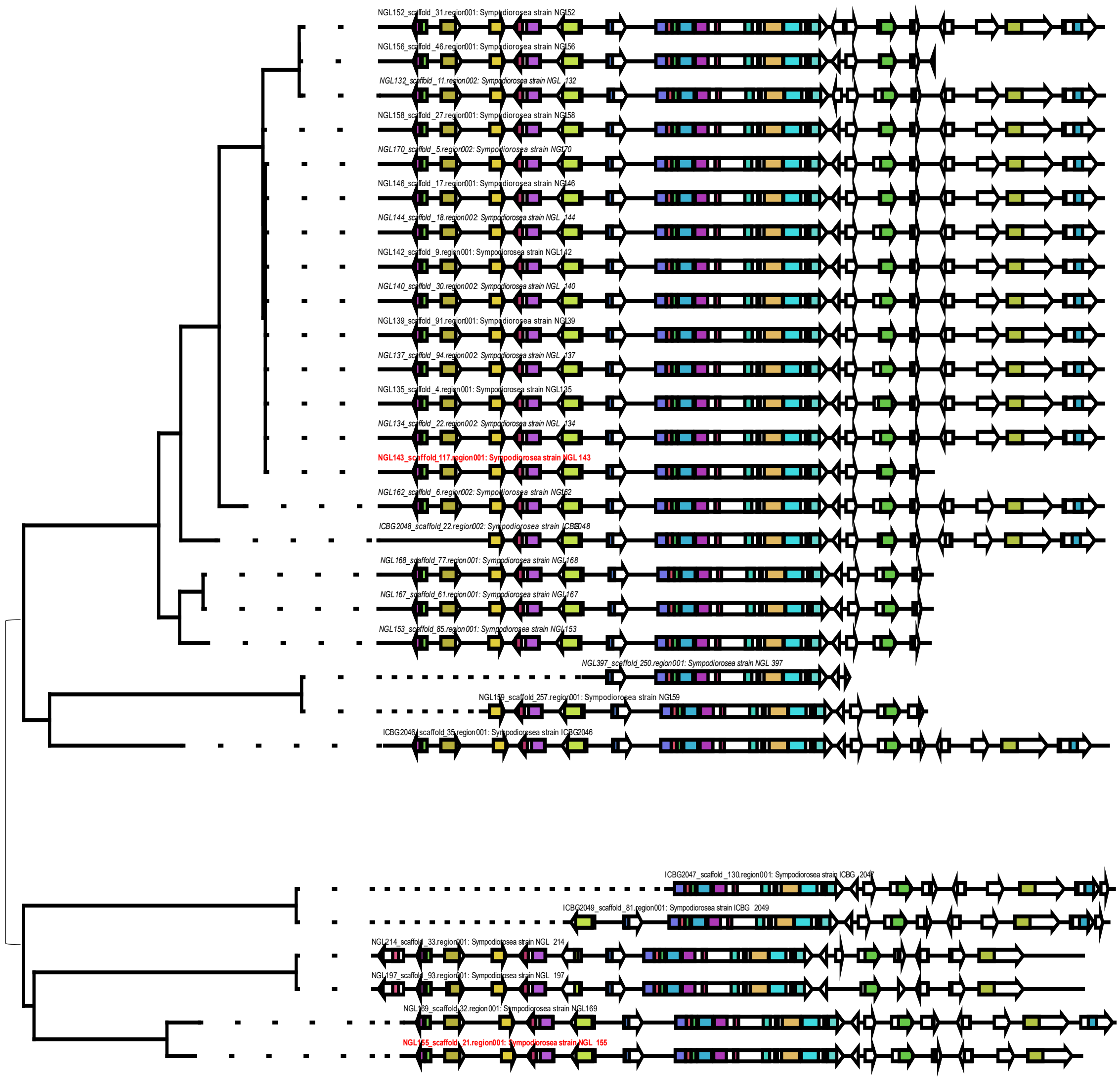

Fig. S12. Emodin analog in *Sympodiorosea*. Alignment and visualization of the emodin analog identified from 28 *Sympodiorosea* genomes sequenced as part of this study. BGC sequences were fragmented for some samples (NGL143, NGL167, NGL168) leading to, at times, incomplete assembly of the BGC for those samples. Additionally, BGC predictions are influenced by the contiguity of genome assemblies leading to, at time, in complete predictions when those clusters are fragmented on different contigs. The Emodin BGC (GCF FAM_00508) was characterized in the sister genus *Escovopsis,* which is obligately associated with higher attine agriculture.

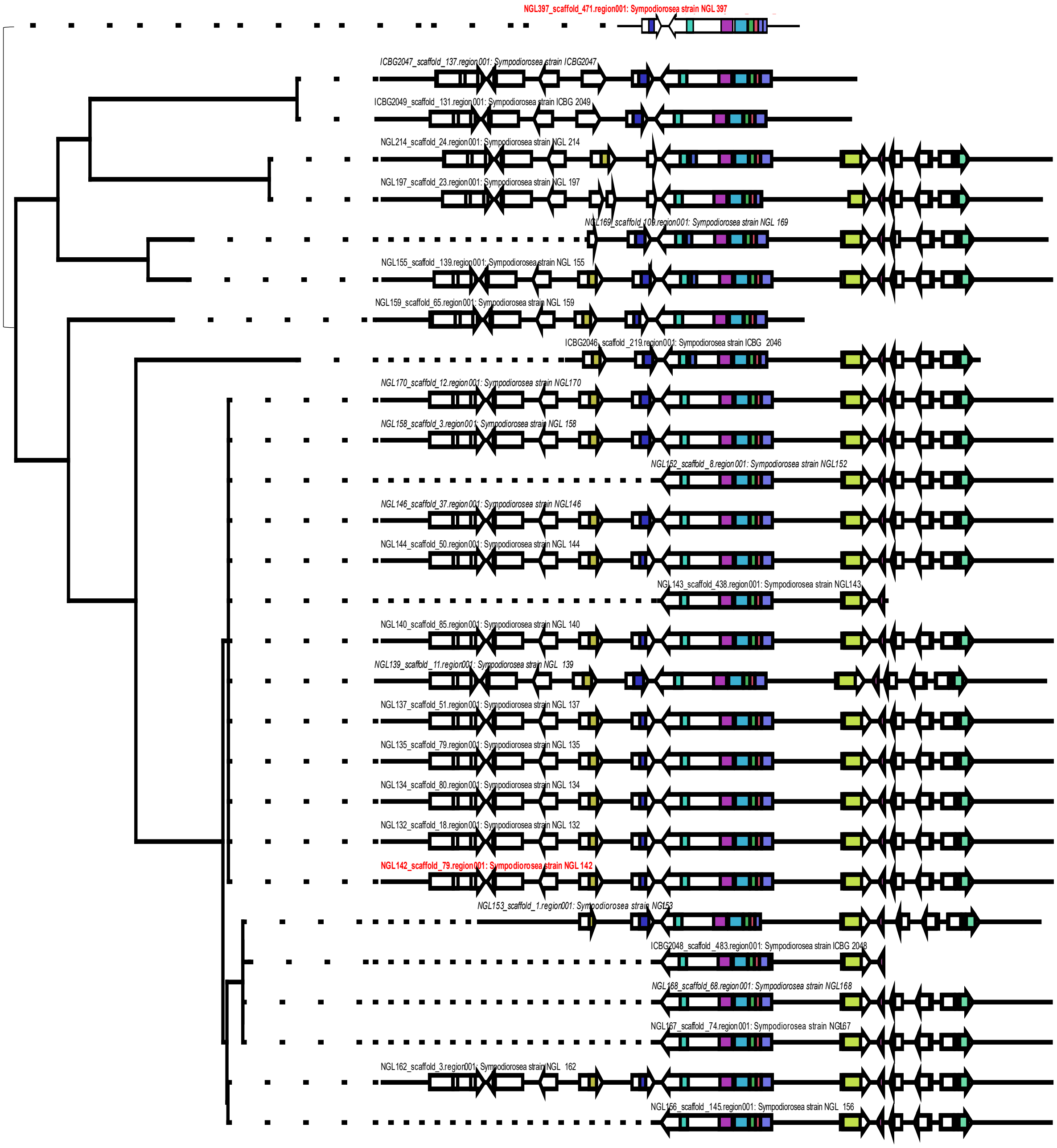

Fig. S13. Shearinine D analog in *Sympodiorosesa*. Alignment and visualization of the Shearinine D analog identified from 27 *Sympodiorosea* genomes sequenced as part of this study. The BGC was absent from sample NGL159. Like the melanicidin IV analog, sequence similarity of this gene was consistent with phylogenomic topology. The melanicidin IV BGC was characterized in the sister genus *Escovopsis,* which is obligately associated with higher attine agriculture.

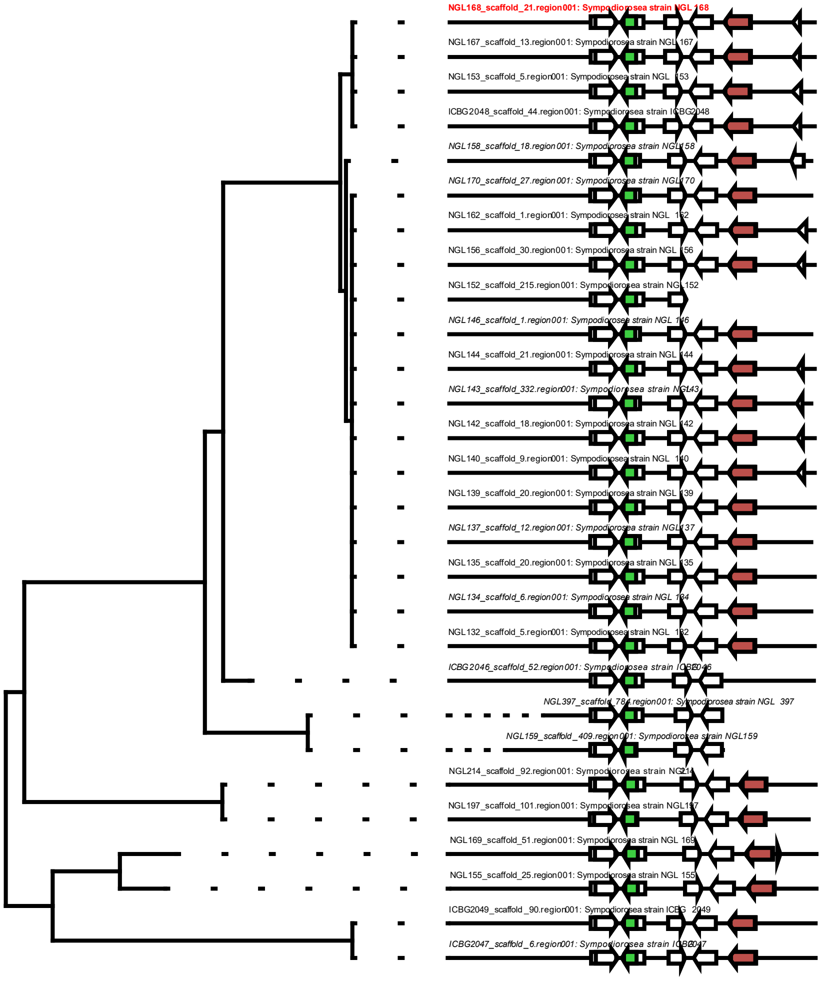

Fig. S14. Test for difference in mean dNdS for metabolism gene annotations and 'background' genomic content. T-test of differences between mean dNdS values indicate that the metabolic gene annotations are under a similar selection regime. Stacked values above the 'NA' or background, are pairwise tests between annotations: bottom, CAZymes (p = 0.03); middle, BGCs (p = 0.99); top, proteases (p = 0.92).

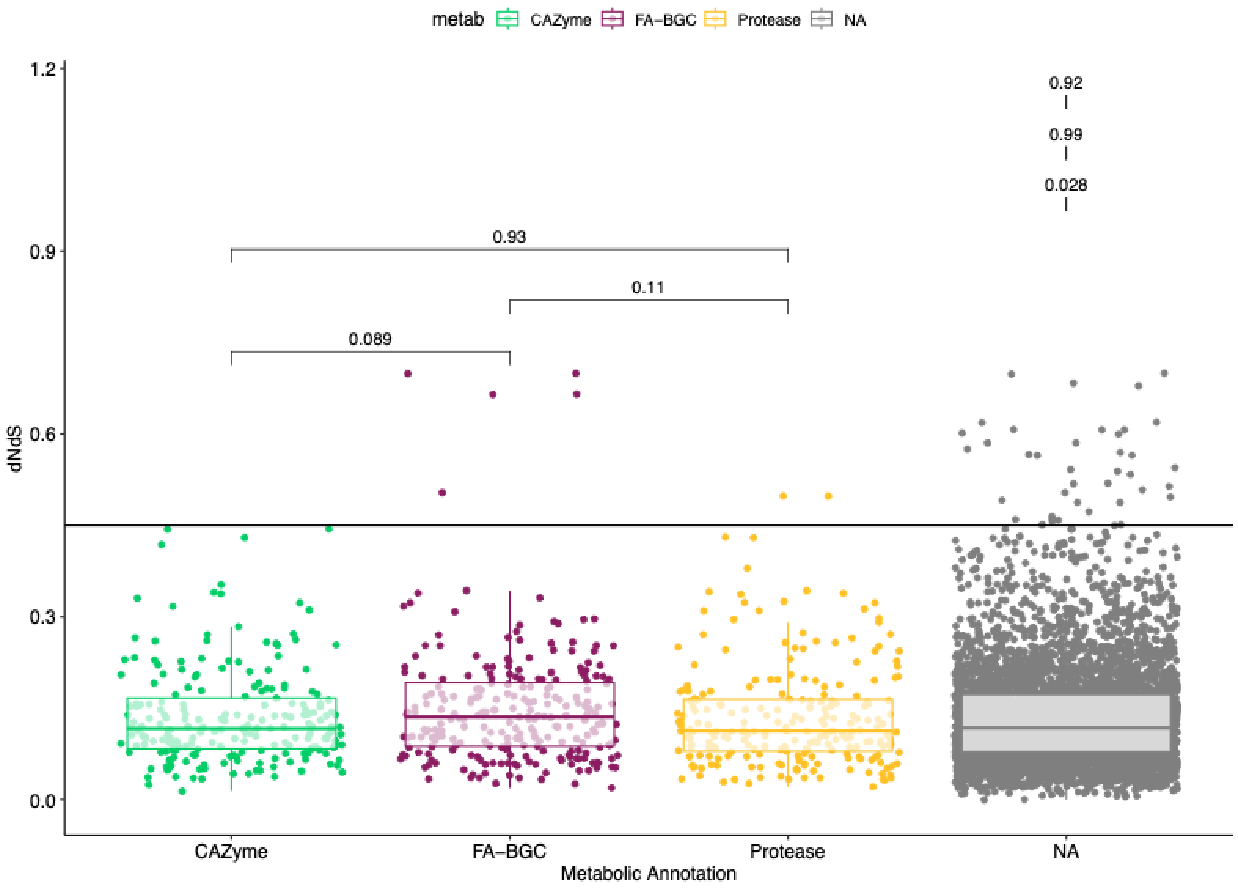

S15. Virulence-associated orthologs with dNdS > 0.45.

Ortholog ID	dN/dS	Putative protein function
orth981	0.699	integral membrane protein related to peroxisome maintenance
orth5915	0.683	zinc finger
orth4371	0.665	Importin-4
orth5968	0.619	GH3
orth5781	0.6	citidyltransferase activity -- virlence in Magnaporthe oryzae
orth5904	0.519	survival motor neuron
orth5383	0.504	hypothetical
orth5813	0.498	extracellular metalloprotease
orth5716	0.497	F-box protein
orth5821	0.491	B-zip transcription factor -- virulence
orth5694	0.472	elongin-a translation factor
orth5377	0.46	telomeric modification/homeobox
orth5603	0.457	transcription regulator zinc finger
orth5841	0.451	host colonization/f-box/transcription regulator
orth5877	0.439	zinc finger
orth5409	0.431	S08A
orth5785	0.43	C48
orth5405	0.423	zinc finger
